# Natalizumab and fumarate treatment differentially modulate CD4+ T cell and B cell subtypes in multiple sclerosis patients without impacting durable COVID-19 vaccine responses

**DOI:** 10.3389/fimmu.2025.1568157

**Published:** 2025-11-19

**Authors:** Ryan Curtin, Yogambigai Velmurugu, Fatoumatta Dibba, Yuan Hao, Samantha Nyovanie, Andrea Lopez, David Mieles, Courtney Ng, Katherine Perdomo, Nicole Scott, James B. Lewin, Robin L. Avila, Jen Smrtka, Yury Patskovsky, Jonathan Howard, Gregg J. Silverman, Michelle Krogsgaard

**Affiliations:** 1 NYU Grossman School of Medicine, New York, NY, United States; 2 NYU Perlmutter Cancer Center, New York, NY, United States; 3 NYU Grossman School of Medicine Department of Pathology, New York, NY, United States; 4 NYU Grossman School of Medicine Applied Bioinformatics Laboratories, New York, NY, United States; 5 NYU Langone Health Multiple Sclerosis Comprehensive Care Center, New York, NY, United States; 6 Biogen, Cambridge, MA, United States; 7 NYU Grossman School of Medicine, Department of Rheumatology, New York, NY, United States

**Keywords:** multiple sclerosis (MS), T cell, B cell, SARS-CoV-2, COVID-19, natalizumab (Tysabri), fumarate, vaccine

## Abstract

**Background:**

There is a greater risk of complications from severe COVID-19 in immunocompromised patients with multiple sclerosis (pwMS) treated with certain disease-modifying therapies (DMTs), as well as a diminished vaccine response.

**Methods:**

In this exploratory, observational study, we recruited 28 patients with Relapsing Remitting MS (RRMS, n=24) or Secondary Progressive MS (SPMS, n=4), that were receiving treatment with either natalizumab or fumarates (diroximel or dimethyl) prior to baseline sample collection. Blood samples were collected before vaccination (baseline), between 4 weeks and 6 months post vaccination, and post booster administration. A multiplex bead immunoassay (MBI) was used to measure anti-Spike IgG, while IFNγ and IL-2 ELISpot assays were used to determine T cell activation. A 35-color spectral flow cytometry panel was used to phenotype bulk B and T cells and SARS-CoV-2-specific T cells, while dimensionality reduction was performed for further phenotypic analysis.

**Results:**

We observed a significantly increased absolute lymphocyte count (ALC) (p=0.0003) in natalizumab-treated pwMS when compared to fumarate-treated pwMS primarily due to increased circulating CD19+ B cells. Fumarate-treated pwMS exhibited a diminished Th1/Th2 ratio when compared to natalizumab-treated pwMS (p=0.0004) or healthy controls (p=0.0745), while natalizumab treatment marginally increased the Th1/Th2 ratio compared to healthy controls (p=0.1311). The observed increase in B cells in natalizumab-treated pwMS were predominantly memory B cells, and double negative (DN) B cells. However, no significant differences between the treatment groups were seen in terms of Spike IgG titers following the initial vaccination course or booster dose, nor in SARS-CoV-2-specific CD4+ responses, all of which remained robust for at least 6 months post-vaccination. The magnitude of humoral and cellular immune responses in both treatment groups were comparable to vaccinated healthy controls. Additionally, SARS-CoV-2 spike-specific CD4+ T cell phenotyping revealed a Th2 dominant response to booster dose in natalizumab-treated pwMS (p=0.0485) but not fumarate-treated pwMS.

**Conclusion:**

pwMS treated with natalizumab or fumarates exhibit similarly robust and durable SARS-CoV-2 specific T cell and humoral responses following vaccination and booster dose. DMT-treated pwMS showing comparable responses to healthy individuals following initial vaccination supports the notion that treatment with these specific DMTs does not diminish strong, long-lasting immunity conferred by COVID-19 vaccination, despite the phenotypic differences modulated by each therapy.

## 1 Introduction

The coronavirus disease 2019 (COVID-19), resulting from severe acute respiratory syndrome coronavirus 2 (SARS-CoV-2), has caused millions of deaths worldwide and is considered an ongoing global pandemic (1, 2). To combat the spread of COVID-19, widespread vaccination efforts were introduced, and the mRNA vaccines BNT162b2 and mRNA-1273 demonstrated the ability to cause robust humoral and cellular-mediated immune responses conferring protection from symptomatic infection in otherwise healthy individuals (3–6). The elderly, obese, and those with diabetes, as well as patients with comorbidities such as autoimmune disorders and various cancers (particularly in those who are treated with immunosuppressive therapies), have been shown to have reduced immune response to vaccination, and an increased risk of severe infection (7–11).

Multiple sclerosis (MS) is an autoinflammatory disease caused by auto-reactive T and B cell infiltration into the CNS resulting in demyelination and progressive neurodegeneration (12–15). As there is no known cure, MS is treated with a series of disease-modifying therapies (DMTs), which have been clinically proven to reduce the occurrence of CNS lesions and slow disease progression. These DMTs work through various mechanisms of action, including immunomodulation (as in the case of dimethyl fumarate and diroximel fumarate), ablation of specific immune cell types (ocrelizumab and cladribine), and inhibiting/altering cellular trafficking (S1P-modulators and natalizumab) (16–19). Many studies have shown that a coordinated humoral and cellular response to vaccination is critical in preventing severe infection (20–25). However, patients with multiple sclerosis (pwMS) treated with some of these DMTs, such as anti-CD20 monoclonal antibodies and S1P receptor modulators, exhibit a reduction in antibodies against the SARS-CoV-2 spike glycoprotein (critical for viral entry into host cells), and/or a reduction in SARS-CoV-2 specific T cell responses, leaving this population of patients at greater risk of severe infection (9, 11, 26).

Natalizumab is a monoclonal antibody against the α4β1-integrin expressed on the surface of circulating lymphocytes which blocks its interaction with vascular cell adhesion molecule 1 (VCAM-1) expressed by vascular endothelial cells, thus reducing lymphocyte migration out of circulation and into the CNS (16). It has been shown that natalizumab treatment results in increased lymphocytes in circulation, but whether this immune modulation results in changes in long-term, antigen-specific memory T cell responses/humoral immune responses is unclear (27, 28). Diroximel/dimethyl fumarate works through several immunomodulatory mechanisms and has been shown to cause a shift towards a more anti-inflammatory, Th2-mediated profile, and additionally by reducing the absolute lymphocyte count (ALC) primarily via depletion of certain populations of inflammatory T cells (18, 29).

While studies have been performed assessing pwMS treated with both natalizumab and diroximel/dimethyl fumarate (referred to henceforth as fumarates) in terms of vaccine responses, most of these studies are not longitudinal and therefore do not address the kinetics of long-term memory T cell and antibody responses following vaccine administration (11, 30–34). Even including the small number of longitudinal studies, no detailed phenotyping data exists for the antigen-specific T cells responding to SARS-CoV-2 vaccination in pwMS treated with natalizumab compared to healthy controls, which is critical to more thoroughly understand its mechanism of action and implication in mRNA vaccine responses. There is also very limited data regarding how pwMS treated with these DMTs respond to a 3rd booster dose, which could further inform on the impact on long-term cellular memory in these patients.

Our study aims to thoroughly characterize the impact of these DMTs up to 6 months after the initial SARS-CoV-2 vaccine series and following a 3rd booster dose. By assessing longitudinal cellular phenotypes in both bulk populations and antigen-specific populations simultaneously, our data provides insights into the potential mechanism of action of natalizumab and fumarate treatment in pwMS both systemically as well as in response to vaccinations. This data furthers our understanding of how these DMTs differentially modulate the immune system, and their implication in the immune response to future vaccinations.

## 2 Methods

### 2.1 Study population

Inclusion criteria: a diagnosis of multiple sclerosis, on a stable dose of diroximel fumarate, dimethyl fumarate, or natalizumab for three months prior to enrollment, and an age of 18–70 years. Patients were excluded if they exhibited a prior or active COVID-19 infection. Statistics describing the patients involved in this study as well as healthy controls are detailed in **Supplementary Tables S1** and **S2**. To thoroughly characterize the cellular and immune response over time in pwMS treated with the disease-modifying therapies (DMTs) fumarates (Tecfidera and Vumerity) or natalizumab (Tysabri), we enrolled 28 total pwMS across multiple time points: pre-vaccine, 4 weeks post-vaccine, 8–12 weeks post-vaccine, 5–6 months post-vaccine, and post-booster. Of the pwMS enrolled, 18 received treatment with natalizumab, and 10 with fumarates. In total, 51 patient samples containing both serum and peripheral blood mononuclear cells (PBMCs), were collected from 28 patients. The number of patients analyzed at each time point is as follows: pre-vaccine (n=6), 4-week post-vaccine (n=5), 8–12 week post-vaccine (n=12), 5–6 month post-vaccine (n=16), post-booster (n=6). The sample distribution by treatment group at each time point is as follows: pre-vaccine included natalizumab (n=6) and fumarate (n=1, excluded due to being the only sample). At 4 weeks post-vaccine, there were natalizumab samples (n=5) and no fumarate samples (n=0). At 8–12 weeks post-vaccine samples included natalizumab (n=9) and fumarate (n=3). At 5–6 month post-vaccine, there were natalizumab (n=11) and fumarate (n=5) samples. Finally, post-booster samples included natalizumab (n=4) and fumarate (n=2). Due to challenges in patient recruitment and sample collection during the ongoing pandemic, not all patients had samples collected at every time point. Specifically, 9 patients provided samples at one time point, 15 patients at two time points, and 4 patients at three time points. At each sample collection visit, patients were routinely asked about COVID-19 infection status and were tested if they exhibited symptoms. While no pwMS had a documented COVID-19 infection before pre-vax sample collection as per the exclusion criteria of the study, two breakthrough infections were reported during the study, both of which were mild to moderate and did not require hospitalization: one fumarate-treated patient at the post-booster timepoint was excluded from analysis due to a lack of viable isolated PBMCs, and one natalizumab treated patient at 6 months post vaccine. Due to difficulties in acquiring longitudinal healthy control samples, healthy controls were only included up to the 8–12-week collection time point. Healthy control samples were provided by the NYU vaccine center. None of the healthy controls had any diagnosed diseases or conditions at the time of sample collection. Due to difficulties with fumarate-treated pwMS enrollment resulting in low patient numbers (n=1 at the pre-vaccination time point and n=0 at the 4-week post-vaccine time point), fumarate-treated pwMS were excluded from these time points.

### 2.2 Analysis of SARS-CoV-2 Spike-specific IgG in patient sera

A proprietary multi-epitope bead-based immunoassay was used to determine the antibody responses toward the SARS-CoV-2 spike protein measured as Spike IgG MFI (mean fluorescence intensity), as previously described (35, 36). Briefly, magnetic beads that were identifiable via internal fluorophores (MagPlex microspheres, Luminex) were coupled to the Wuhan variant total spike protein (Sino Biological cat no. 40590-V08B). For controls, tetanus toxoid, human serum albumin, and anti-human IgG were also coupled with beads and utilized within each assay performed (Jackson immunoresearch). The MagPix platform (Luminex) was used for detection of SARS-CoV-2 spike protein binding.

### 2.3 SARS-CoV-2 Spike peptide library

For use in the stimulation of PBMC before ELISpot and flow cytometry assays, a commercially available SARS-CoV-2 peptide library containing reconstituted lyophilized 15-mer peptides covering the surface of the entire spike glycoprotein with 11 amino acid (aa) overlap was used (Miltenyi Biotec PepTivator ^®^ SARS-CoV-2 Prot_S Complete, cat. 130-129-712).

### 2.4 Analysis of cellular responses to SARS-CoV-2 spike protein

Commercially available Enzyme-Linked immunospot (ELISpot) kits were used to quantify interferon-gamma (IFNγ) and interleukin-2 (IL-2)-secreting cells following stimulation with the previously described SARS-CoV-2 spike peptide library using a protocol that was described previously (35, 37). ELISpot kits were purchased from Cellular Technology Limited (CTL, hIFNγ ELISpot pre-coated and hIL-2 ELISpot pre-coated, cat. hIFNγ−p-1M/5 and hIL2p-1M/5, respectively). Briefly, 100,000 PBMC per well were incubated with either spike peptide library at 2 μg/mL final concentration, Phytohemagglutinin (M form) (Gibco, cat. 10576-015) at a final concentration of 1.5% as a positive control, or media alone as a negative control, for 48 hours. The S6 Universal M2 analyzer was used for reading the plates (CTL), and assay readouts were calculated as spot-forming units (SFU) per 1e6 cells.

### 2.5 Antibody panel

A comprehensive, 35-parameter spectral flow cytometry panel was generated specifically for use with the Cytek Aurora Spectral Analyzer (Cytekbio). Details of the panel including markers and fluorophores can be found in **Supplementary Table S3**.

### 2.6 B and T cell phenotyping

The 35-parameter antibody panel was utilized for the detection of bulk B and T cells, and incorporated Activation Induced Markers (AIM) for assessment of SARS-CoV-2 spike-specific T cells. To account for non-specific background T cell activation that can be seen in some pwMS, an unstimulated, media-only condition was included for every patient in addition to the stimulated condition using the previously described SARS-CoV-2 peptide pool. Approximately one million PBMCs per patient were thawed into complete RPMI media supplemented with 5% human serum, centrifuged, and washed in PBS before being resuspended in FACS buffer consisting of Hanks balanced salt solution (HBSS) supplemented with 2% fetal bovine serum (FBS). Fc receptor blocking was performed using the Human TruStain FcX and True-Stain Monocyte Blocker (BioLegend cat. 422302 and cat. 426102, respectively) for 5 minutes at room temperature. 5 μL of each reagent was mixed with FACS buffer for incubation. Antibody staining was performed in a final volume of 50 μL per sample for 30 minutes at 22°C, followed by 2 washes with HBSS and subsequent resuspension in Zombie NIR™ Fixable Viability dye (BioLegend cat. 423106) in HBSS. After another wash in HBSS, a fixation/permeabilization step was performed according to the manufacturer’s recommendations using the eBioscience™ Foxp3/Transcription Factor Staining Buffer Set (ThermoFisher Scientific, cat. 00-5523-00). 1X permeabilization buffer was used for the following two washes. 1X permeabilization buffer was then used to dilute the intracellular antibodies outlined in **Supplementary Table S3**, and intracellular staining was performed for 1 hour at 4°C. After another 2 washes in the permeabilization buffer, cells were resuspended in FACS buffer described previously. As mentioned, data acquisition was performed using the Cytek Aurora Spectral Analyzer combined with the proprietary Cytek SpectroFlo software version 3.2.1.

### 2.7 Analysis of spectral flow cytometry data

FlowJo v10 (BD Biosciences) was used to analyze cell phenotypes acquired for each patient sample using the gating strategies for bulk B cells (**Supplementary Figure S1**), and bulk T cells (**Supplementary Figures S8**, **S9**). AIM+ cells were identified and the same gating strategies in **Supplementary Figures S8** and **S9** were applied for AIM+ cell phenotyping (38). For downstream analysis, files were exported in CSV format. Graphs were made using GraphPad Prism version 9.0.0.

### 2.8 High-dimensional data processing

CSV files containing channel values exported from FlowJo were first converted into FCS format using the flowCore package (39) available in R/Bioconductor (40, 41) and stored as a SingleCellExperiment object (42, 43). For computational efficiency, a subset of all events (100K by default) was randomly selected for downstream analysis. To ensure representative events were selected from all samples of all batches, but without introducing additional sample bias, the total number of events was first divided equally among all batches and evenly distributed among samples within each batch. Batch correction was performed using the ComBat program (44, 45), followed by dimension reduction and Louvain clustering (46). Uniform Manifold Approximation and Projection (UMAP) (47) was chosen for cluster visualization.

### 2.9 Statistical analysis

**Figure 1:**
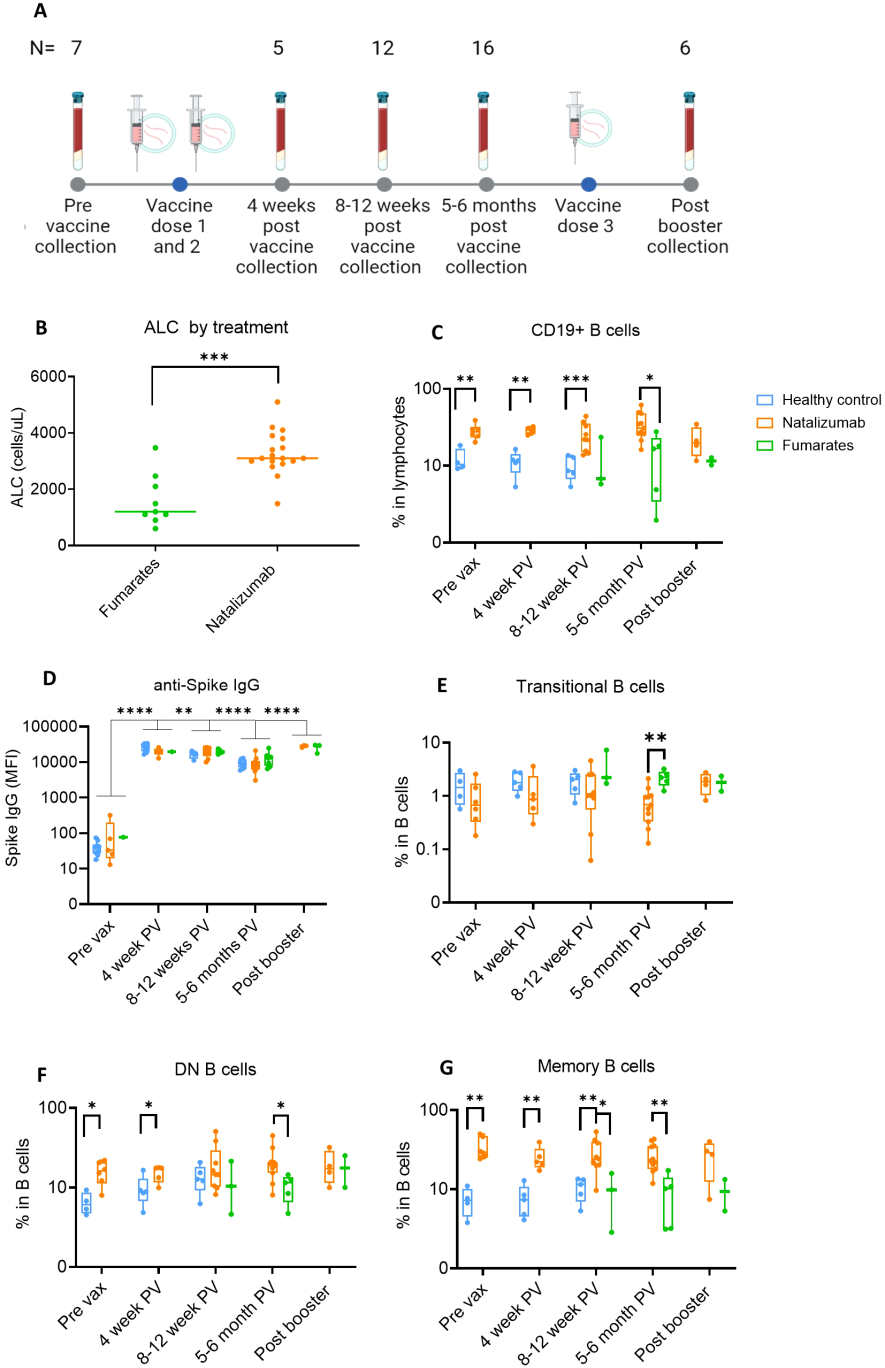
Anti-Spike IgG levels in natalizumab and fumarate-treated pwMS are comparable to healthy controls up to 6 months of SARS-CoV-2 mRNA vaccination. **(A)** Collection timeline for natalizumab and fumarate-treated pwMS, including post-booster collection timepoint. The pre-vaccine baseline sample was collected before the initial two-dose vaccine series. The number of patient samples at each time point is shown above each time point, the breakdown of each treatment at each time point can be found in methods. **(B)** Comparison of Absolute Lymphocyte Count (ALC) between fumarate green) and natalizumab (orange) treated pwMS at the pre-vaccine collection timepoint, measured as cells/uL. **(C)** Comparison of %CD19+ B cells in lymphocytes between healthy control (blue), natalizumab treated pwMS (orange), and fumarate-treated pwMS (green), across all analyzed pre- and post-vaccination collection time points. **(D)** Anti-Spike IgG levels as determined by MBI assay, displayed as Mean Fluorescence Intensity (MFI), compared between healthy control (blue), natalizumab treated pwMS (orange), and fumarate-treated pwMS (green). **(E-G)** % Transitional B cells **(E)**, Double Negative (DN) B cells **(F)**, and Memory B cells **(G)**, compared between all collected time points, plotted as the percentage of each B cell subtype in total B cells. Healthy control (blue), natalizumab treated pwMS (orange), and fumarate treated pwMS (green) are shown. Mann-Whitney test was used for statistical comparisons in all panels except **(D)**, where a 2-way ANOVA with Benjamini, Krieger and Yekutieli two-stage step up method to control for False Discovery Rate was used p<0.05 (*), p<0.01 (**), p<0.001 (***), and p<0.0001 (****).

GraphPad Prism version 9.0.0 and version 9.1.2 were used to perform statistical analysis. Patient data was not normally distributed. The Mann-Whitney test (non-parametric) was used to compare different patient groups within specific time points. A Kruskal-Wallis test with Benjamini, Krieger and Yekutieli two-stage step up method to control for False Discovery Rate was used to compare a single treatment group across multiple time points. A 2-way ANOVA with Benjamini, Krieger and Yekutieli two-stage step up method to control for False Discovery Rate was used to assign significance when comparing responses to vaccination between all treatments and all measured time points (this was utilized specifically to compare column and row effect in the Spike IgG production data in **Figure 1D**). As this is an exploratory study with a small sample size (particularly at the post booster time points), the Mann-Whitney test was used to assign significance when comparing the 5–6 month post-vaccine timepoint to the post booster time point in the context of SARS-CoV-2 Spike-specific T cell responses. Statistical significance was defined as a value of p <.05 (*). Non-significant (NS) differences were denoted by the absence of p-values. Additional statistical significance levels include p<.01 (**), p<.001 (***), and p<.0001 (****). Additionally, we performed a statistical power analysis on our cohort utilizing the baseline blood draws for each patient and their respective absolute lymphocyte counts (ALC). Sample sizes were n=9 for fumarates (1 patient did not have ALC data) and n=18 for natalizumab. A Wilcoxon Rank Sum Test with a 2-sided alpha of.05 was used to determine that with this sample size, differences between the groups are detectable with 98% power. For variations measured at multiple time points post-baseline, a Mann-Whitney test was used. Based on these sample sizes, with +/- 1.5 standard deviations and a two-sided alpha = 0.5, we had 80% power to detect changes from baseline. No adjustments were made for multiple statistical tests due to the varying recruitment of patients at each time point.

## 3 Results

### 3.1 Natalizumab increases memory B cell subtypes while maintaining robust and durable anti-Spike IgG responses to initial SARS-CoV-2 vaccination and booster in pwMS

To first compare the ALC between each treatment group, we used the baseline blood draw from each patient, as this is when the clinical demographics for the pwMS were collected (**Supplementary Table S1**, healthy controls in **Supplementary Table S2**). The longitudinal collection timeline is shown in **Figure 1A**. ALC was significantly higher (p=.0003) in natalizumab-treated compared to fumarate-treated pwMS (**Figure 1B**). This effect has been reported previously when compared to both healthy controls and fumarate-treated pwMS and is likely to occur due to a natalizumab-induced increase in to % CD19 B cells in total lymphocytes (**Figure 1C**) (48–50). We first measured the percentage of CD19 B cells and thoroughly characterized the impact of natalizumab treatment on memory and other circulating B cell subtypes (**Supplementary Table S3**). The gating strategy for B cell subset identification is described in **Supplementary Figure S1**. We observed in all analyzed pwMS that natalizumab-induced increase in B cell content did not result in any significant difference in anti-Spike IgG production following two initial vaccine doses or booster dose when compared to fumarate-treated pwMS or healthy controls (**Figure 1D**). Anti-Spike IgG levels declined significantly between the 4-week and 8–12-week post-vaccine time point (p=.0067) and between the 8-12-week post-vaccine time point and the 5–6-month post-vaccine time point (p=<.0001) but increased following booster (p=<.0001) (**Figure 1D**). There were no differences in Spike IgG levels between either DMT when compared to healthy controls at any of the measured time points, supporting the idea of robust, long-term anti-spike humoral immunity in these pwMS (**Figure 1D**).

While previous literature has indicated that natalizumab can result in a higher number of circulating memory B cells, memory B cell subsets and their distribution in these pwMS were not investigated (49). We observed a significant reduction in the proportion of transitional B cells between fumarate- and natalizumab-treated pwMS at 5–6 months post-vaccine (**Figure 1E**). The most striking differences in B cell subtype frequency between treatments were present among memory B cells, both in IgD- CD27- atypical memory or double negative (DN) B cells, and CD27+ CD38- classical classical memory B cells (**Figures 1F, G**). Compared to healthy controls, natalizumab-treated pwMS had significantly higher percentages of classical memory B cells at pre-vaccine (p=.0095), 4 weeks post-vax (p=.0079), and 8–12 weeks post-vax (p=.0070), as well as compared to fumarate-treated pwMS at 8–12 weeks post vax (p=.0364) and 5–6 months post-vax (p=.0018) (**Figure 1G**). Because both DN and classical memory B cells can be divided into several functionally distinct cell types, we decided to further investigate these subsets in our study population (51).

We observed no differences between healthy controls, fumarate-treated pwMS, or natalizumab-treated pwMS (**Supplementary Figures S2A-C**) when the relative proportions of each DN B cell subset were compared by treatment group. DN1 and DN3 B cells comprised a majority of DN B cells in circulation (**Supplementary Figures S2A-C**). Furthermore, the proportions of switched memory (sM), IgD-only memory (dM), unswitched memory (usM), and IgM-only memory (mM) B cell subsets were unaffected by treatment with either natalizumab or fumarates and were comparable to healthy controls (**Supplementary Figures S2D-F**). Our data indicate that natalizumab significantly bolsters the percentage of CD27+ CD38- memory B cells in circulation without altering the balance of memory B cell subtypes compared to healthy controls or fumarate-treated pwMS. Irrespective of these changes in B cell phenotype, natalizumab-treated pwMS exhibit a robust and durable anti-Spike IgG response that is stable up to 6 months post-vaccination and further increased by a 3rd booster dose in natalizumab-treated pwMS.

### 3.2 Unbiased phenotyping of CD19+ B cells in natalizumab-treated pwMS reveals differences in the naïve and switched memory compartments compared to healthy controls and fumarate-treated pwMS

**Figure 2:**
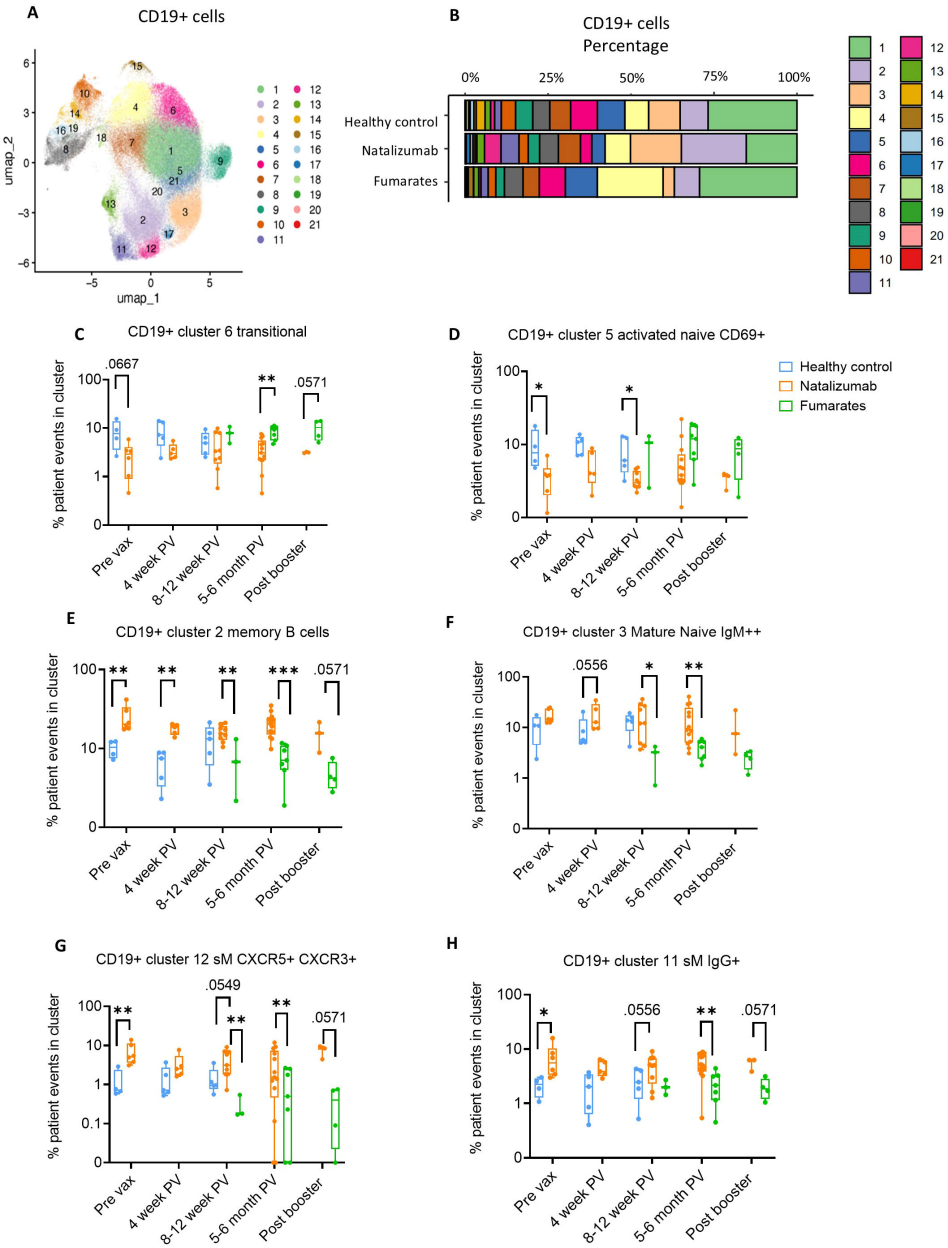
Memory B cell composition is differentially impacted by natalizumab treatment compared to fumarate treatment in pwMS. **(A)** UMAP depicting all CD19+ B cells collected from all pre- and post-vaccine collection time points, including both fumarate and natalizumab-treated pwMS for phenotyping purposes. 21 clusters were identified for further analysis. **(B)** The proportion of each identified CD19+ B cell cluster compared between healthy controls, natalizumab treated pwMS, and fumarate treated pwMS. **(C-H)** Longitudinal comparison of phenotyped UMAP clusters showing transitional B cells **(C)**, CD69+ activated naïve B cells **(D)**, CD27+ classical memory B cells **(E)**, IgM+ mature naïve B cells **(F)** CXCR5+ CXCR3+ switched memory (sM) B cells **(G)**, and IgG+ sM B cells **(H)** between healthy control (blue), natalizumab treated pwMS (orange) and fumarate treated pwMS (green). Each dot represents a single patient, plotted as the percentage of events in a particular cluster out of the total number of events collected for that patient. Mann-Whitney test was used for statistical comparisons. P<0.05 (*), p<0.01 (**) and p<0.001 (***).

Uniform manifold approximations and projections (UMAP)s were created for each treatment group at each time point for unbiased comparison of B cell phenotypes between healthy controls, natalizumab-treated pwMS, and fumarate-treated pwMS (**Supplementary Figures S3A-C**). The combined UMAP used for phenotyping is shown in **Figure 2A**, with the proportions of each cluster in **Figure 2B**.

To further characterize B cell subsets in these pwMS after dimensionality reduction, we performed clustering based on surface marker expression to characterize B cell phenotypes in an unsupervised manner. The heatmap and table of cell surface markers used for CD19+ B cell phenotyping are shown in **Supplementary Figures S4A, B**. As seen in our traditional gating analysis of the flow cytometry data, there was a significant decrease in the percentage of transitional B cells in natalizumab-treated pwMS compared to fumarate-treated pwMS at 5–6 months post-vaccine (p=.0032) (**Figure 2C**). Compared to healthy controls, there was also an observable trend towards lower percentages of transitional B cells pre-vax (p=.0667) (**Figure 2C**). In addition to validating our flow cytometry-based observations, UMAP projections of total CD19+ B cells also provided a more comprehensive look at the landscape of cellular markers used within our panel, thus allowing for more detailed phenotyping of B cell subsets.

Since mature naïve B cells can exhibit various states of differentiation and activation and therefore display different fates upon antigen encounter, we examined naïve B cell clusters that were further differentiated from each other by the expression of various differentiation/maturation and activation markers, such as CD69 and CXCR5 (52). While CD69+ activated mature naïve B cells were significantly decreased in natalizumab-treated pwMS compared to healthy controls at pre-vax and 8–12 weeks post-vax (p=.0381 and p=.010, respectively), an opposite trend was observed among the non-activated, IgM_high_ mature naïve B cells, especially when compared to fumarate treated pwMS (**Figures 2D–F**). Since it has been established that IgM+ mature naïve B cells will migrate to secondary lymphoid organs and undergo class switch recombination (CSR) after antigen encounter (52), we analyzed if there was a change in switched memory B cells. Indeed, CD27+ IgD- IgM- CXCR3+ CXCR5+ switched memory B cells were significantly increased in natalizumab-treated pwMS compared to fumarate-treated at 8–12 weeks post-vaccine (p=.0182) and 5–6 months post-vaccine (p=.0090) with a similar trend compared to healthy controls (p=.0556) (**Figure 2G**). Additionally, the cluster of CD27+ IgD- IgM- IgG+ sM B cells was also significantly increased in natalizumab-treated pwMS, compared to both fumarate-treated at 8–12 weeks post vaccine (p=.0091) and healthy controls pre-vaccination (p=.0095) (**Figure 2H**). These observed changes demonstrate a systemic effect of natalizumab treatment resulting in the increased circulating percentage of memory B cells, particularly of switched memory B cells (**Figures 2E–H**). Our data supports the idea that despite treatment-induced changes in the proportion of B cell subsets when comparing natalizumab and fumarate-treated pwMS, robust humoral responses to SARS-CoV-2 Spike antigen are present for at least 6 months following the initial vaccine series.

### 3.3 Fumarate treatment results in an anti-inflammatory shift defined by decreased CD4+ effector memory T cells and increased Th2 cells when compared to natalizumab treated pwMS

Multiple studies have indicated an impact of natalizumab treatment on both circulating CD4+ and CD8+ T cell phenotypes; particularly naïve, central memory, and effector memory (27, 28) However, longitudinal analysis of these phenotypes during consistent treatment with natalizumab and fumarates has not been performed following vaccination. Using the antibody panel described in **Supplementary Table S2**, we characterized both CD4+ and CD8+ bulk T cell phenotypes in this cohort of pwMS by spectral flow cytometry. The assessment of bulk T cell populations is critical in determining if any systemic phenotypic shifts induced by natalizumab or fumarate treatment underly changes in the activation and phenotype of SARS-CoV-2 specific T cells following vaccination, as it has been shown that the most effective immune response against COVID-19 following vaccination is defined not just by humoral immunity, but by a coordinated humoral and cellular immune response (20).

**Figure 3:**
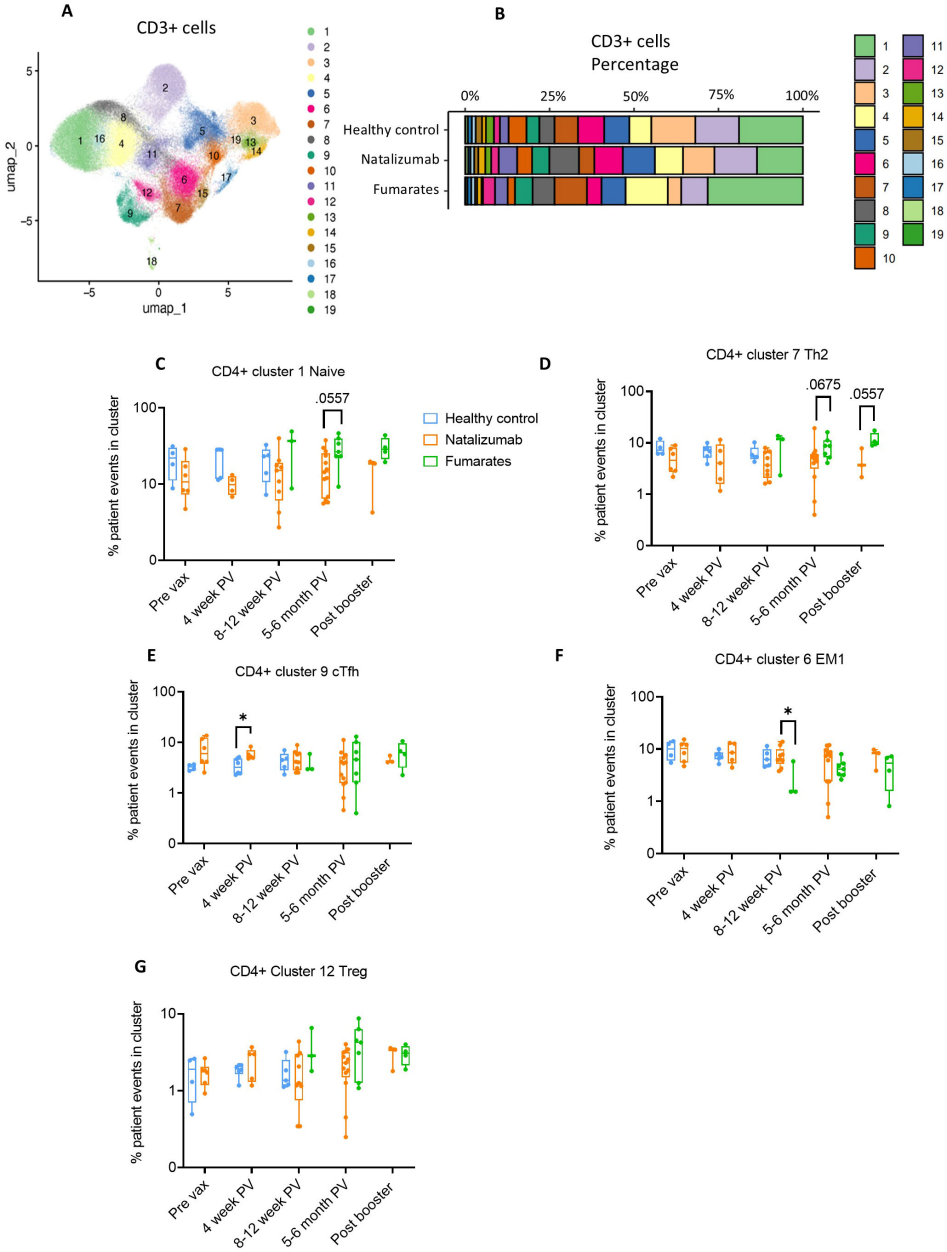
Fumarate treatment results in a shift towards an anti-inflammatory CD4+ T cell composition when compared to natalizumab treatment in pwMS. **(A)** UMAP depicting all CD3+ T cells collected from all pre- and post-vaccine collection time points, including both fumarate and natalizumab-treated pwMS for phenotyping purposes. 19 clusters were identified for further analysis. **(B)** The proportion of each identified CD3+ T cell cluster compared between healthy controls, natalizumab treated pwMS, and fumarate treated pwMS. **(C–G)** Longitudinal comparison of phenotyped UMAP clusters showing naïve T cells **(C)**, Th2 T cells **(D)**, CXCR5+ circulating T follicular helper (cTfh) T cells **(E)**, effector memory 1 (EM1) T cells **(F)**, and T regulatory T cells **(G)** between healthy control (blue), natalizumab treated pwMS (orange) and fumarate treated pwMS (green). Each dot represents a single patient, plotted as the percentage of events in a particular cluster out of the total number of events collected for that patient. Mann-Whitney test was used for statistical comparisons. P<0.05 (*).

Similarly to CD19+ B cell analysis, we performed dimensionality reduction on the CD3+ T cells from each patient and created UMAP projections for fumarate-treated pwMS, natalizumab-treated pwMS, and healthy controls to analyze bulk T cell phenotypes in an unbiased manner (**Supplementary Figures S5A-C**). The heatmap and a table outlining cellular markers used to phenotype bulk CD3+ clusters are shown in **Supplementary Figures S6A, B**. From the traditional flow gating, we compared the CD4/CD8 ratio and Th1/Th2 ratio between treatments using each patient’s baseline blood draw, as these ratios provide insight into immune cell homeostasis as well as inflammatory vs. anti-inflammatory skewing within immune cells (**Supplementary Figures S6C–F**). Fumarate-treated pwMS exhibited a significant increase in bulk %CD4+ compared to natalizumab-treated pwMS (p=.0027) and healthy controls (p=.0111) as well as a significant decrease in bulk %CD8 compared to natalizumab treated pwMS and healthy controls (p=.0040, p=.0206, respectively), resulting in a significant increase in CD4/CD8 ratio compared to healthy controls (p=.0206) and natalizumab-treated pwMS (p=.0039) (**Supplementary Figures S6C-E**). Natalizumab-treated pwMS and healthy controls had similar CD4/CD8 ratios (**Supplementary Figure S6E**). This was in parallel with an observed decrease in bulk Th1/Th2 ratio following fumarate treatment, compared to both healthy controls (p=.0745) and natalizumab treated pwMS, (p=.0004), demonstrating skewing towards anti-inflammatory immune cell phenotype following fumarate treatment in these pwMS (**Supplementary Figure S6F**). Based on the data shown in **Figure 3A**, proportions of patient cells in each cluster separated by treatment were calculated and used to determine clusters of interest for further analysis (**Figure 3B**). First, we analyzed bulk CD8+ T cell clusters, where no significant differences were observed between natalizumab or fumarate-treated pwMS when compared to healthy controls in any of the examined CD8+ clusters (**Supplementary Figures S7A-D**). Consistent with the fumarate-induced shift towards a more anti-inflammatory immune cell composition, we observed a significant decrease in CD8+ EM1 cells in fumarate-treated pwMS when compared to natalizumab treated pwMS at 8–12 weeks post-vaccine (p=.0182), and a similar trend at the post booster time point (**Supplementary Figure S7D**). When examining CD4+ T cells, a strong increasing trend in CD4+ naïve (p= .0557) and Th2 T cells (p=.0675) was observed in pwMS treated with fumarates compared to natalizumab, particularly at the 5–6-month post-vaccine time point which contained the most patient samples (**Figures 3C, D**). Minimal changes in bulk T cell memory phenotypes were caused by natalizumab treatment when compared to healthy controls, with no statistically significant differences being observed in CD4+ circulating T follicular helper (cTfh), CD4+ effector memory 1 (EM1), or CD4+ regulatory T cells (Treg) (**Figures 3E–G**). Our data shows that CD4+ and CD8+ immune cell phenotypes are largely preserved following natalizumab treatment when compared to healthy controls.

. Taken together, our phenotyping study indicates that fumarate treatment results in a shift towards an anti-inflammatory T cell composition marked by a significant decrease in bulk Th1/Th2 ratio and the trend towards a decrease in CD4+-EM1 compared to natalizumab treated pwMS. Our data reveals the effect of these DMTs by demonstrating specific alterations in memory T cell subsets such as a reduction in EM1 T cells following fumarate treatment.

### 3.4 SARS-CoV2-specific CD4+ memory T cells are present in natalizumab and fumarate-treated pwMS at least 6 months following SARS-CoV-2 vaccination

**Figure 4:**
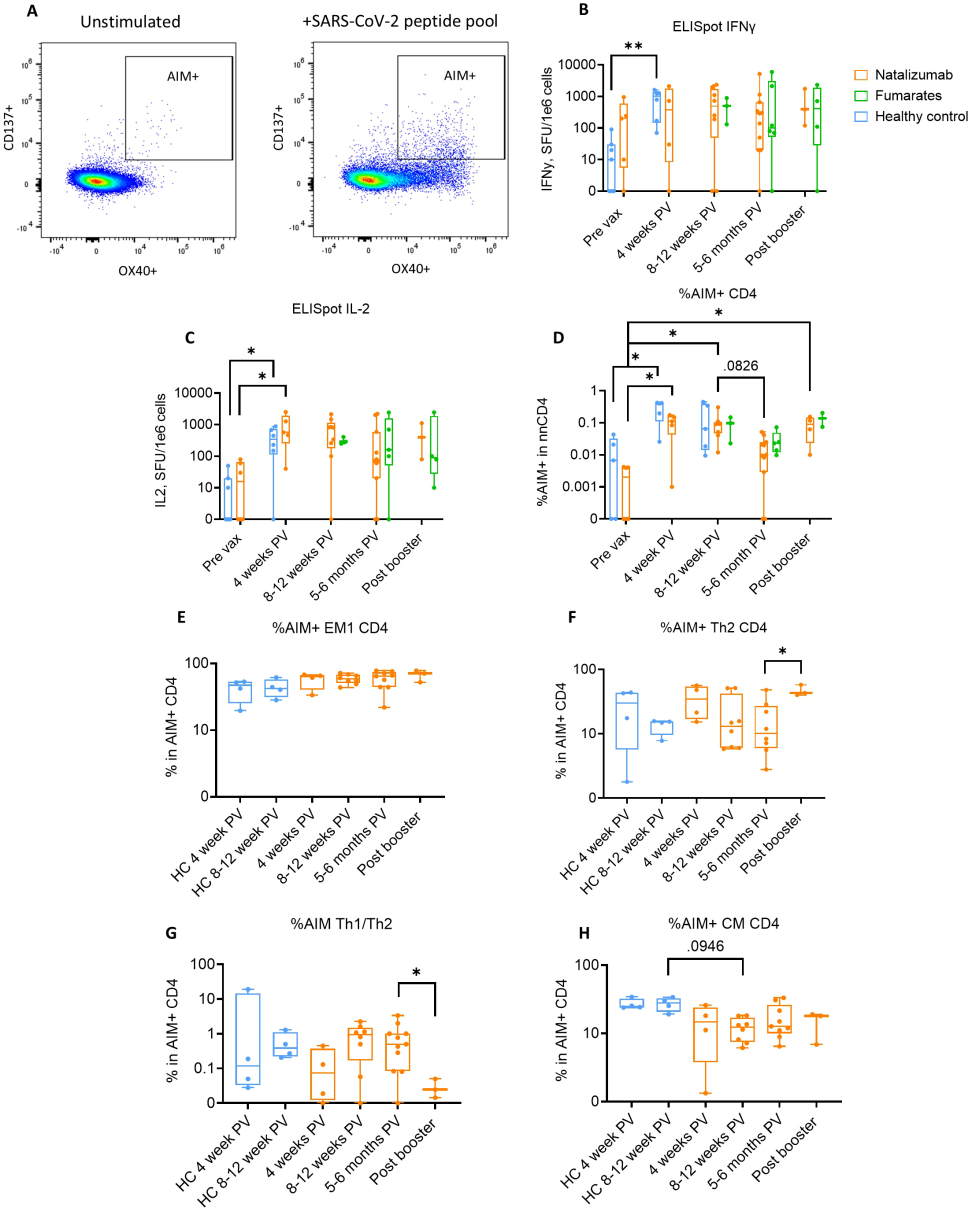
Natalizumab-treated pwMS demonstrate a robust and durable SARS-CoV-2 spike-specific CD4+ T cell response that is comparable to healthy controls up to 4 months after SARS-CoV-2 vaccination. **(A)** Gating strategy used for identification of activation-induced marker (AIM) positive cells. AIM+ CD4+ T cells were defined as CD137+OX40+. The left panel shows unstimulated T cells (media only), while the right panel shows T cells from the same patient following stimulation for 24 hours with a SARS-CoV-2 spike peptide pool. **(B, C)** Longitudinal ELISpot assays measuring IFNγ **(B)** and IL-2 **(C)** spot-forming units (SFU) per 1e6 cells following 48-hour stimulation with SARS-CoV-2 spike peptide pool. Healthy control (blue), natalizumab treated pwMS (orange), and fumarate treated pwMS (green) are shown. **(D)** Longitudinal comparison of %SARS-CoV2 spike specific AIM+ CD4+ T cells in non-naïve CD4+ T cells (nnCD4) between healthy control (blue), natalizumab treated pwMS (orange), and fumarate treated pwMS (green) across all collected timepoints. **(E–H)** Comparison of identified AIM+ CD4+ T cell phenotypes between healthy controls (blue) and natalizumab treated pwMS (orange). Each AIM+ phenotype is plotted as a percentage of the total number of AIM+ CD4+ T cells from each patient. Displayed phenotypes include EM1 **(E)**, Th2 **(F)** Th1/Th2 ratio (**G**, calculated by dividing the percentage of Th1 AIM+ cells by the percentage of Th2 AIM+ cells), and central memory (CM) **(H)**. A Kruskal-Wallis test with Benjamini, Krieger and Yekutieli two-stage step up method to control for False Discovery Rate was used to compare a single treatment group across multiple time points. A Mann-Whitney test was used for statistical comparisons between the 5–6 months post-vaccine and post booster time points due to low sample size. P<0.05 (*) and p<0.01 (**).

We then evaluated the SARS-CoV-2 specific T cell responses in pwMS using IFNγ or IL-2 ELISpot analysis. SARS-CoV-2 spike-specific AIM+ cells were determined by dual expression of CD137 and CD134 (**Figure 4A**). While no significant increase in the number of spike-specific T cells was seen at 4 weeks post-vaccine, the robust IFNγ response to spike peptide library was detected and sustained at other time points and up to 6 months post-vaccine in both natalizumab and fumarate-treated pwMS as compared to healthy controls (**Figure 4B**). A significant increase in antigen specific memory T cells was observed following vaccination in healthy controls (p=.0023) (**Figure 4B**).

A similar trend was observed in the IL-2 ELISpot assay, but we observed a statistically significant increase in IL-2 secreting spike-specific T cells following vaccination in natalizumab-treated pwMS (p=0.0075) (**Figure 4C**). Our findings indicate that drug-treated pwMS exhibit a response in magnitude comparable to healthy controls following vaccination with a durability of up to 6 months, independent of DMT administration. To gain a better understanding of what T cell phenotypes were primarily responding to SARS-CoV-2 spike peptide stimulation, we examined the relative abundance of CD4+ AIM+ (CD137+ OX40+) non-naïve CD4+ (nnCD4) population (**Figure 4D**). Due to the low number of enrolled fumarate-treated pwMS and low number of AIM+ CD4+ cells from those patients, only natalizumab-treated pwMS were compared to healthy controls. The gating strategies used for the phenotyping of AIM+ CD4+ T cells are shown in **Supplementary Figures S8** and **S9**. We also examined AIM+ CD8+ T cell responses, which are shown in **Supplementary Figure S10**. Compared to healthy controls, natalizumab-treated pwMS showed a similarly significant increase in %AIM+ CD4+ T cells at 4 weeks post-vaccination (p=.0194) and 8–12 weeks post-vaccination (p=.0041) when compared to pre-vaccine (**Figure 4D**). Additionally, there was a strong and significant correlation between IFNγ ELISpot responses and %AIM+ nnCD4+ cells at 4 weeks post-vaccine, but not at baseline, in all pwMS regardless of treatment (**Supplementary Figures S10A–C**). This correlation was present at 5–6 months post-vaccine as well but did not reach statistical significance. Natalizumab-treated pwMS showed a near-significant reduction in CD4+ AIM+ cells at 5–6 months post-vaccination compared to the previous time point (p=.0826) but, following booster dose, the increased proportion of these cells was comparable to that seen following the initial vaccination (p=.0378) (**Figure 4D**). Despite this observed decrease at 5–6 months post-vaccination, the difference between SARS-CoV-2 S stimulated patient CD4+ T cells and their unstimulated counterparts were significant at all post-vaccine time points, indicating that a strong T cell response is still ongoing (**Supplementary Figure S10D**). No differences between fumarate-treated or natalizumab-treated pwMS were observed at 8–12 weeks post-vaccine, 5–6 months post-vaccine, or post booster dose in ELISpot or AIM assays. The concordant data from both ELISpot and AIM assays indicate that both natalizumab and fumarate-treated pwMS form a robust, antigen-specific T cell response to the initial vaccine series and the booster dose that is comparable to healthy controls, showing that the vaccine response is persevered in these pwMS despite DMT administration.

### 3.5 Natalizumab-treated pwMS show differences in antigen-specific CD4+ T cell phenotypes compared to healthy controls

Next, we aimed to thoroughly characterize the CD4+ AIM+ T cell phenotypes in natalizumab-treated pwMS compared to healthy controls, given the established importance of effector memory cells in SARS-CoV-2 vaccine responses (20). Due to difficulties in phenotyping extremely low cell numbers, patients with fewer than 11 AIM+ cells were excluded (pre-vax time point n=6 and one natalizumab-treated post-booster patient). The antigen-specific EM1 response was robust in natalizumab-treated pwMS and comparable to healthy controls up to 12 weeks post-vaccine, indicating a strong effector memory response is not impacted by natalizumab treatment (**Figure 4E**). As we observed a trend towards an increase in bulk Th1/Th2 ratio in natalizumab treated pwMS compared to healthy controls, we analyzed SARS-CoV-2 Spike-specific Th1 and Th2 CD4+ cells, and their kinetics over time. We observed a significant increase in %AIM+Th2 cells following booster dose administration (p=.0485) (**Figure 4F**). This increase was not present in fumarate-treated pwMS at the same time points (**Supplementary Figures S10E, F**). To account for shifts in both AIM+ Th1 and AIM+ Th2 populations, we looked at the Th1/Th2 ratio of AIM+ CD4+ T cells only. Interestingly, a significant (p=.0357) reduction in the Th1/Th2 ratio in natalizumab treated pwMS following booster dose was also observed (**Figure 4G**). In the CD4+ AIM+ CM compartment, natalizumab treated pwMS showed a lower percentage among total CD4+ AIM+ cells at 8–12 weeks post-vaccine when compared to healthy controls (p=.0946) (**Figure 4H**). The increased CD4+ AIM Th2 response to the booster dose did not coincide with any changes in the CD8+ AIM+ EM1 responses following the booster dose, which closely resembled the responses observed in the CD4+ AIM+ EM1 subtype (**Supplementary Figure S10G**). CD8+ AIM+ CM T cell responses to the initial vaccine series and booster dose also similarly mirrored the responses seen in CD4+ AIM+ CM T cells, with a significant decrease at 8–12 weeks post-vaccine in natalizumab treated pwMS compared to healthy controls (p=.0028) (**Supplementary Figure S10H**). Together, these observations suggest that the natalizumab-mediated increase in CD4+ AIM+ Th2 responses to booster dose does not impact these antigen-specific CD8+ AIM+ memory T cell subsets (**Supplementary Figure S10H**). In conclusion, our data suggests that EM1 responses are unaffected by natalizumab treatment, while AIM+ Th2 cells appear to be dominant over AIM+ Th1 responses to the booster dose in natalizumab-treated pwMS. Taken together, our data show that natalizumab treatment does result in changes in both bulk B/T cell phenotypes and antigen-specific T cell responses to SARS-CoV-2 vaccination, but that these changes do not result in deficits in these patients’ overall humoral or cellular responses to vaccination.

## 4 Discussion

The use of DMTs in the treatment of MS carries the additional risk of altering the immune cell populations that confer protection against severe SARS-CoV-2 infection, even following vaccination. For this reason, it is critical to understand the impact of these MS therapies in the context of SARS-CoV-2 vaccine responses. In this study, we thoroughly characterized the impact of natalizumab and fumarate treatment on circulating bulk B and T cell phenotypes in pwMS, as well as assessed the humoral and cellular response to SARS-CoV-2 vaccination up to 6 months after the primary vaccine course and following the third booster dose. Using several assays including spectral flow cytometry, ELISpot, and a proprietary bead-based multi-epitope immunoassay (MBI), DMT-treated pwMS were compared to healthy controls up to 6 months for humoral responses, and up to 3 months for cellular responses.

Previous research has observed that the ALC of pwMS treated with natalizumab was significantly increased compared to healthy controls as well as other DMTs, primarily due to an increase in circulating B cells, which was confirmed in our patient cohort (48, 50). Additionally, it has been shown that natalizumab and fumarate-treated pwMS mount a significant humoral immune response to SARS-CoV-2 mRNA vaccination, although healthy controls were not included in the study and the impact of a 3rd booster dose was not assessed (30). Sabatino et al. similarly assessed pre- vs. post-vaccination cellular and humoral immune responses in pwMS receiving multiple DMT including natalizumab, and also observed robust responses to SARS-CoV-2 vaccination, however only pre-vaccine and approximately 4-week post-vaccine time points were compared (26). Our study both analyzes the impact of natalizumab and fumarate treatment on the systemic B and T cell subpopulations in pwMS as well as provides an in-depth phenotypic characterization of the SARS-CoV-2 antigen-specific T cells in natalizumab treated pwMS for up to 6 months following vaccine administration and post-booster dose. This data addresses missing links in the mechanism of action of these DMTs with regards to mRNA vaccine responses as well as having implications for response to viral infections.

Several interesting findings emerged from our study. We observed a robust humoral immune response comparable to that of healthy controls at 4 weeks post-vaccine, which declined significantly up to 5–6 months post-vaccination. This has been observed before in otherwise healthy patients, but had not yet been thoroughly examined in MS patients treated with natalizumab or fumarates (53). Critically, the humoral response to booster dose was not impacted by either treatment and was comparable to healthy controls. After phenotyping all B cell subsets in circulation, we observed that in addition to an increase in CD19+ CD27+ CD38 mid/low B cells in natalizumab-treated pwMS, there was a significant increase in DN B cells in comparison to both fumarate-treated pwMS and healthy controls There is debate regarding the various DN B cell subsets and their roles in immune responses in healthy controls as well as in patients with autoimmune disorders ranging from production of pathogenic autoantibodies to serving as antigen-experienced atypical memory B cells differentiating without the use of germinal centers, as well as acting as T cell co-stimulators and antigen presenting cells (22, 54). For this reason, we also analyzed the DN1, DN2, and DN3 subset proportions representing the total DN B cell population. We observed no differences in the relative proportions of each DN B cell subset between different treatments, suggesting that the systemic increase in DN B cells is tied to the overall increase in memory B cells seen in natalizumab-treated pwMS. This further suggests that natalizumab may increase circulating memory B cells involved in extrafollicular humoral response in addition to the canonical germinal center response seen from classical memory B cells (22). Given the wide array of surface markers utilized in our antibody panel, we were able to assess multiple memory B cell subsets that have yet to be analyzed in natalizumab or fumarate-treated pwMS. An interesting dichotomy was seen between activated naïve CD19+ CD38mid/low IgD+ CD69+ B cells, which were significantly reduced in natalizumab-treated pwMS compared to healthy controls when compared to mature naïve CD19+ CD38mid/low IgD+ IgM+ B cells, which showed a significant increase in natalizumab-treated pwMS. There was observed significant increase in two clusters of switched memory B cells as compared to healthy controls and fumarate treated pwMS. A possible explanation for this is that mature naïve IgM+ B cells are serving as a pool of B cells preferentially selected to migrate to secondary lymphoid organs, where they would then differentiate into sM B cells (52). Despite these differences in memory B cell populations, there was no observable difference in anti-spike IgG responses as measured by MBI; responses among natalizumab- and fumarate-treated pwMS closely mirrored those of healthy controls up to 6 months post-vaccination. Although healthy control data was lacking at the post-booster timepoint, a significant increase in anti-spike IgG was still present in both DMT-treated pwMS compared to the previous timepoint, overall supporting the idea of robust and durable humoral immune responses in both natalizumab and fumarate treated pwMS.

While a previous study by Gross et al. observed a significant increase in the percentage of CD4+ Th2 cells following fumarate treatment in pwMS, our results comparing the percentage of CD4+ Th2 cells in fumarate-treated pwMS to natalizumab-treated pwMS and to healthy controls, and showed only statistically insignificant increases compared to natalizumab-treated pwMS, and no difference compared to healthy controls (29). However, this statistical insignificance does not rule out the idea that fumarate treatment results in a shift towards a more anti-inflammatory state. At 8–12 weeks post-vaccine, we also observed a significant decrease in the percentage of CD4+ effector memory 1 (EM1) and CD8+ EM1 in fumarate-treated-pwMS-compared-to natalizumab-treated pwMS, which further supports the idea that fumarate treatment results in less inflammatory immune cell composition. Interestingly, a small but significant increase in the proportion of circulating T follicular (cTfh) in natalizumab treated pwMS compared to healthy controls was present. Since this T cell population is critical for interaction with B cells to drive humoral immune responses, this observation is likely due to the increased number of B cells in circulation in natalizumab-treated pwMS that would necessitate greater crosstalk between the humoral and cellular arms of the immune response in these patients (55).

A unique strength of our study is the in-depth phenotyping of the SARS-CoV-2 antigen-specific T cell response to mRNA vaccination in natalizumab-treated pwMS, using both ELISpot assays for cells secreting IL-2 and IFNγ in response to peptide library stimulation as well as a spectral flow cytometry-based AIM assay. Interestingly, we observed a significant increase in IL-2-secreting cells, but not IFNγ- secreting cells, at 4 weeks post-vaccination. This is likely due to background activation of T cells secreting IFNγ at the pre-vaccine time point, which could be due to inflammation inherent to the MS phenotype, or cross-reactivity with other seasonal coronaviruses (56–58). Even so, ELISpot for both cytokines showed a robust and durable antigen-specific T cell response, with no significant decline in responding cells up to 6 months post-vaccination in pwMS treated with either fumarates or natalizumab. To circumvent any background activation, we performed the AIM assay and subtracted the background activation from the stimulated samples using the unstimulated control sample for each patient analyzed. The AIM assay indicated a similarly robust and durable antigen-specific CD4+ response, which was comparable between pwMS treated with either DMT and healthy controls. Due to the more sensitive nature of this assay compared to ELISpot, we observed a decrease in antigen-specific T cells at 5–6 months post-vaccination, followed by a significant increase in response to booster. Further validating our findings is the significant correlation that was observed between ELISpot responses and AIM assay responses at post-vaccination time points.

Lastly, when evaluating the phenotypes of the SARS-CoV-2 antigen-specific T cells in natalizumab treated pwMS, we observed a significant increase in %CD4+ Th2 cells responding to the booster dose. This represents a novel finding, as systemically, natalizumab has been shown to increase the Th1/Th2 ratio compared to other DMTs and skew the immune response towards a Th1/pro-inflammatory state (27). The increased Th1/Th2 ratio in natalizumab treated pwMS compared to fumarate-treated pwMS was also present in our study. A recent study examined SARS-CoV-2 antigen-specific CD4+ T cells in pwMS treated with multiple DMTs and reported that natalizumab showed the greatest percentage of antigen-specific CD4+ Th0/Th2 cells compared to healthy controls and other DMT-treated pwMS at a median of approximately 4 months post most recent vaccination (38). Together, these observations support the idea of a Th2 dominant immune response to booster dose in natalizumab treated pwMS, despite the elevated percentage of bulk CD4+ Th1 cells at baseline resulting from this treatment. However, no differences in EM1, shown in previous literature to be the dominant responding subtype to SARS-CoV-2 vaccination including in pwMS, were observed, supporting the idea that natalizumab treatment does not impact effector memory responses to vaccination (9, 59). When we analyzed AIM+ CD8+ EM1 T cell responses to SARS-CoV-2 vaccination, we found that they closely resembled the responses seen in the AIM+ CD4+ EM1 T cell subsets, and the antigen-specific CD8+ EM1 booster dose response was not impacted by the Th2-dominant response seen in AIM+ CD4+ T cells in natalizumab-treated pwMS. Further studies are necessary to determine the exact nature of this Th2 dominant response and its implication in the mechanism and function of natalizumab treatment in these pwMS. Additionally, we observed a reduction of antigen-specific CD4+ central memory T cells at 8–12 weeks post-vaccination in natalizumab treated pwMS compared to healthy controls, which was mirrored by the AIM+ CD8+ CM T cell response. It is unclear whether this reduction would persist as healthy control data for further time points are lacking. As antigen-specific CM T cells are more likely than effector memory cells to be re-activated in secondary lymphoid organs and remain there to aid in B cell differentiation, we postulate that the overall increase in memory B cell subsets in natalizumab treated pwMS could facilitate the increased migration of CM T cells out of circulation and into these secondary lymphoid organs for this purpose (60).

The impact of breakthrough infections on humoral and cellular immune responses is of great importance when evaluating longitudinal data following SARS-CoV-2 vaccination, in both healthy subjects and in pwMS. Previous literature observes that humoral immunity, particularly through expansion of the memory B cell subset, is enhanced following breakthrough infection (61). In our study, however, only two breakthrough infections were reported, and those patients were excluded from analysis, giving our study a unique angle that allows for analysis of purely vaccine responses across a 6 month time period post-vaccination. While it is possible that asymptomatic infections may have occurred during this time period, we still observe that humoral and cellular immune responses to vaccination are comparable to healthy controls regardless of treatment with natalizumab or fumarates in pwMS.

We recognize that our study is not without limitations. Difficulties in patient recruitment (dropouts) resulted in a small sample size at the earliest timepoints post-vaccination, as well as a lack of fumarate patients for the SARS-CoV-2 antigen-specific phenotyping. Our dataset is also not perfectly longitudinal, as some patients had sample collections at one, two, or three timepoints, but not at each timepoint. The lack of availability of post-booster healthy controls is another limitation, however, we were still able to compare natalizumab- and fumarate-treated patients at the post booster time point despite the small sample size. Additional studies with more patient samples will be critical to further investigate the Th2 dominant immune response to SARS-CoV-2 booster dose in natalizumab-treated pwMS, as this is a novel finding that could have clinical implications regarding the mechanism of action of this DMT.

Taken together, our findings provide insights into the impact of natalizumab treatment on the antigen-specific CD4+ T cell response to vaccinations and provide evidence of changes in antigen-specific T cell subsets caused by this DMT. Our data shows the preservation of antigen-specific CD4+ memory T cell subpopulations as well as increases in memory B cells, supporting the idea that administration of natalizumab does not diminish protection against severe SARS-CoV-2 infection. These findings support the idea of robust and durable CD4+ cellular memory as well as humoral immune responses to the initial SARS-CoV-2 vaccination and booster dose. Additionally, our findings contribute to the understanding of the mechanism of action of natalizumab as far as its impact on T and B cell subpopulations known to be involved in the defense against severe SARS-CoV-2 infection.

## Data Availability

The raw data supporting the conclusions of this article will be made available by the authors, without undue reservation.
