# Supplementary material for: Natalizumab and fumarate treatment differentially modulate CD4+ T cell and B cell subtypes in multiple sclerosis patients without impacting durable COVID-19 vaccine responses
Source: Front Immunol. 2025 Nov 19;16:1568157. doi: 10.3389/fimmu.2025.1568157 (PMC12672258; doi:10.3389/fimmu.2025.1568157)
Supplement: Supplementary file 1 [file DataSheet1.pdf]

Table 1: Clinical and Demographic Features (n=28)	
**Age, years**	
Mean ± SD (median, range)	45.90 ± 13.25 (48.8, 18-68)
**Sex, n (%)**	
Female	20 (71.4%)
Male	8 (28.6%)
**Race, n (%)**	
White	12 (42.9%)
Black	13 (46.4%)
Other/Unknown	6 (21.4%)
**Ethnicity, n (%)**	
Hispanic or Latino	7 (25%)
Non-Hispanic or Latino	21 (75%)
**MS Type, n (%)**	
Relapsing-Remitting	24 (85.7%)
Secondary Progressive	4 (14.3%)
**Current DMT, n (%)**	
Natalizumab (Tysabri)	18 (64.3%)
Diroximel Fumarate (Vumerity)	5 (17.9%)
Dimethyl Fumarate (Tecfidera)	5 (17.9%)
**Smoking Status, n (%)**	
Current Smoker	4 (14.3%)
Former Smoker	5 (17.9%)
Non-smoker	5 (17.9%)
**Clinically Significant Diseases, n (%)**	
Hypertension	3 (10.7%)
Asthma	4 (14.3%)
Cardiovascular disease	2 (7.1%)
Diabetes mellitus	2 (7.1%)
Rheumtologic/Immunologic Disease	1 (3.6%)
Gastrointestinal Disease	3 (10.7%)
Other Neurologic Disease	2 (7.1%)
Other	9 (32.1%)
None	13 (46.4%)
**Vaccine type , n (%)**	
Pfizer	23 (82%)
Moderna	5 (18%)

Supplementary Table 1

	Healthy control
**Number of patients (n)**	9
**Age (mean, range)**	44.2, 35
**Race (%)**	
White:	67%
Black	11%
Other	22%
**MS subtype (%)**	
RRMS	N/A
SPMS	N/A
**Vaccine type (%)**	
Pfizer	56%
Moderna	44%
**Sex (%)**	
Male	44%
Female	56%

Supplementary Table 2

Marker	Fluorophore	Manufacturer
Live/Dead	LiveDead Blue	Fisher
CD3	SparkBlue 550	BioLegend
CD4	SparkViolet 538	BioLegend
CD8	PE-Fire 640	BioLegend
CD11c	PerCP	BioLegend
CD134(OX40)	BUV805	BD Biosciences
CD24	BV480	BD Biosciences
CD19	BUV496	BD Biosciences
CD137(4-1BB)	A647	BioLegend
CD21	PE-Cy5	BD Biosciences
CD23	BUV615	BD Biosciences
CD25	BUV563	BD Biosciences
CD27	SB702	Invitrogen
CD38	APC/Fire810	BioLegend
CD40	PacBlue	BioLegend
CD45RA	Spark NIR 685	BioLegend
IgM	BV570	BioLegend
CCR6	SB780	Invitrogen
CD69	BV650	BioLegend
CD127	SparkYG 581	BioLegend
CD138	BV510	BioLegend
CD95	BV421	BioLegend
CD183 (CXCR3)	BV750	BD Biosciences
CD185 (CXCR5)	BB515	BD Biosciences
CD197 (CCR7)	BV605	BioLegend
CD278 (ICOS)	APC-Fire750	BioLegend
CD279 (PD-1)	BB700	BD Biosciences
HLA-DR	BUV661	BD Biosciences
IgD	BUV737	BD Biosciences
Foxp3	PE-Cy5.5	Invitrogen
Tbet	PE-Cy7	BioLegend
CD40L	PE-eF610	Invitrogen
GzmB	A700	BD Biosciences
CD194(CCR4)	BUV395	BD Biosciences
IgG (as surface marker)	PerCP-Vio700	Miltenyi

Supplementary Table 3

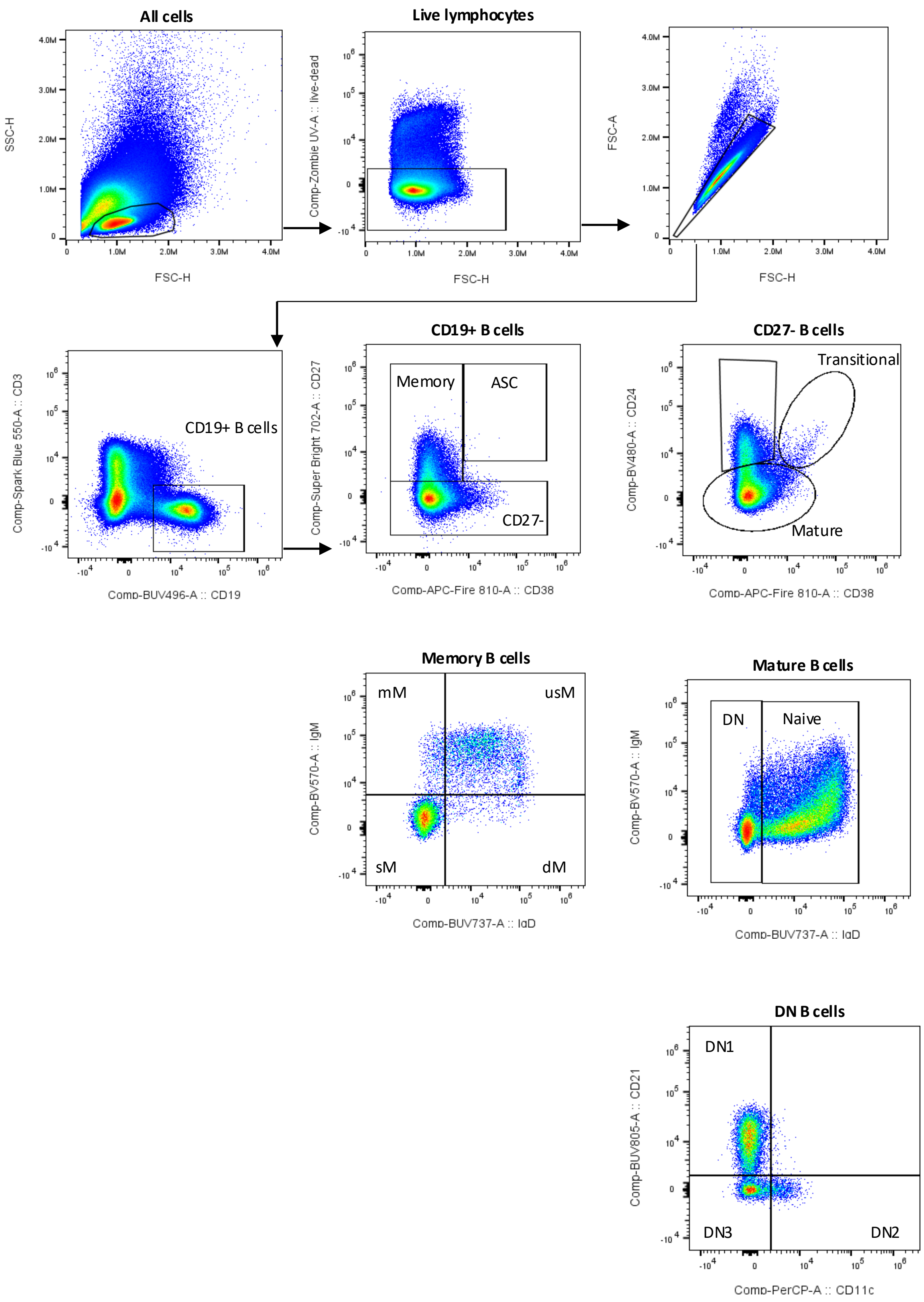

Supplementary Figure 1

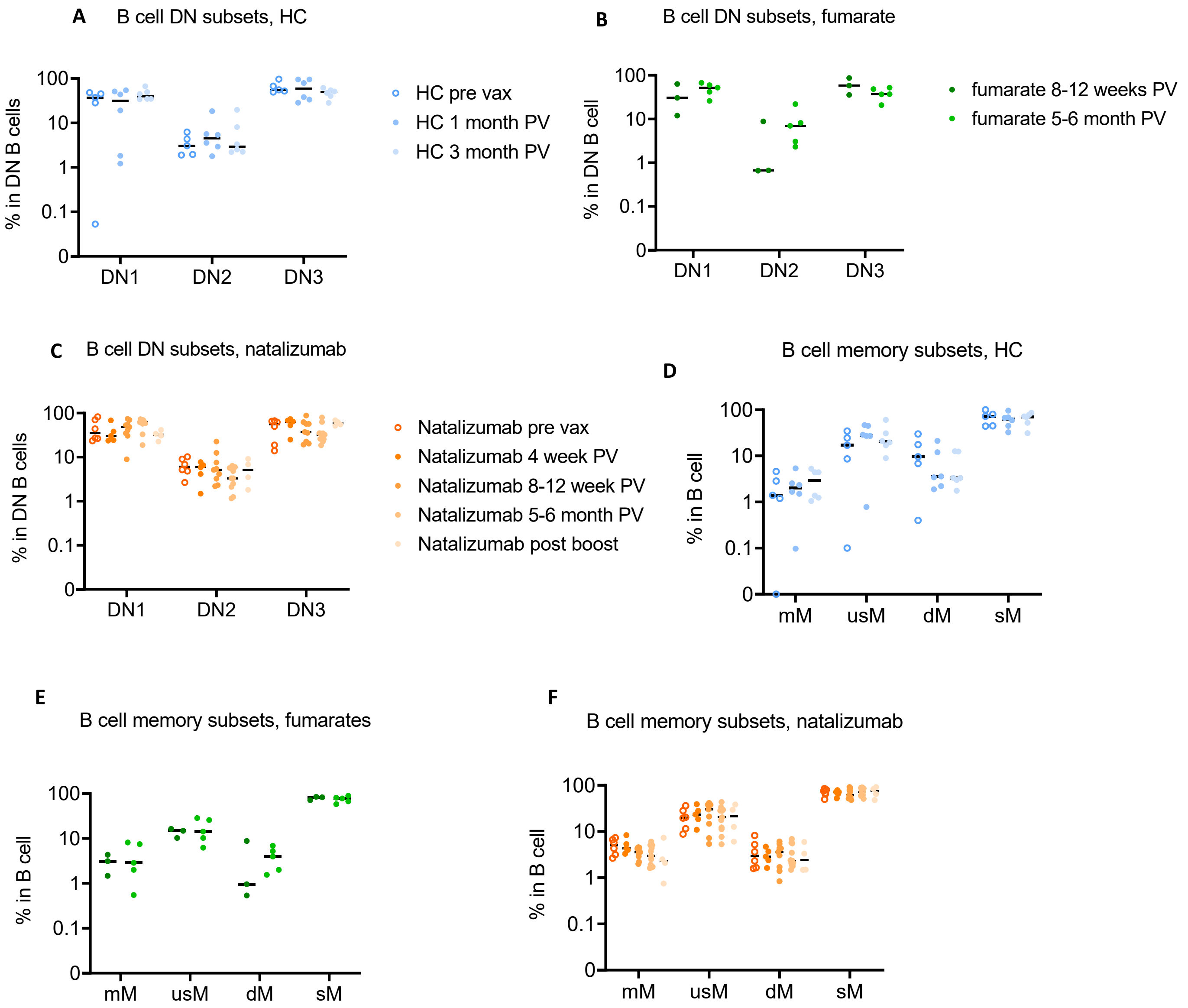

Supplementary Figure 2

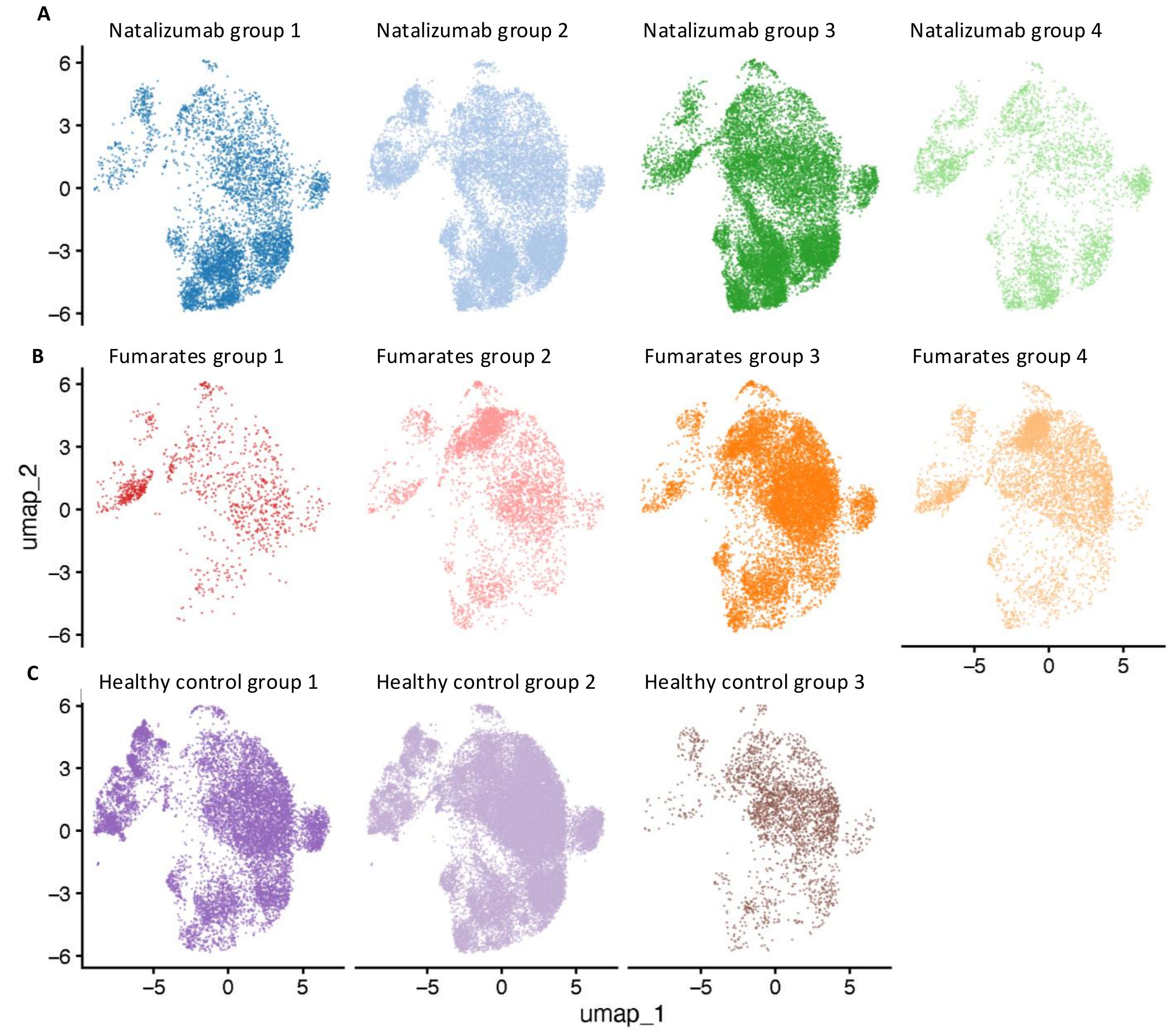

Supplementary Figure 3

A

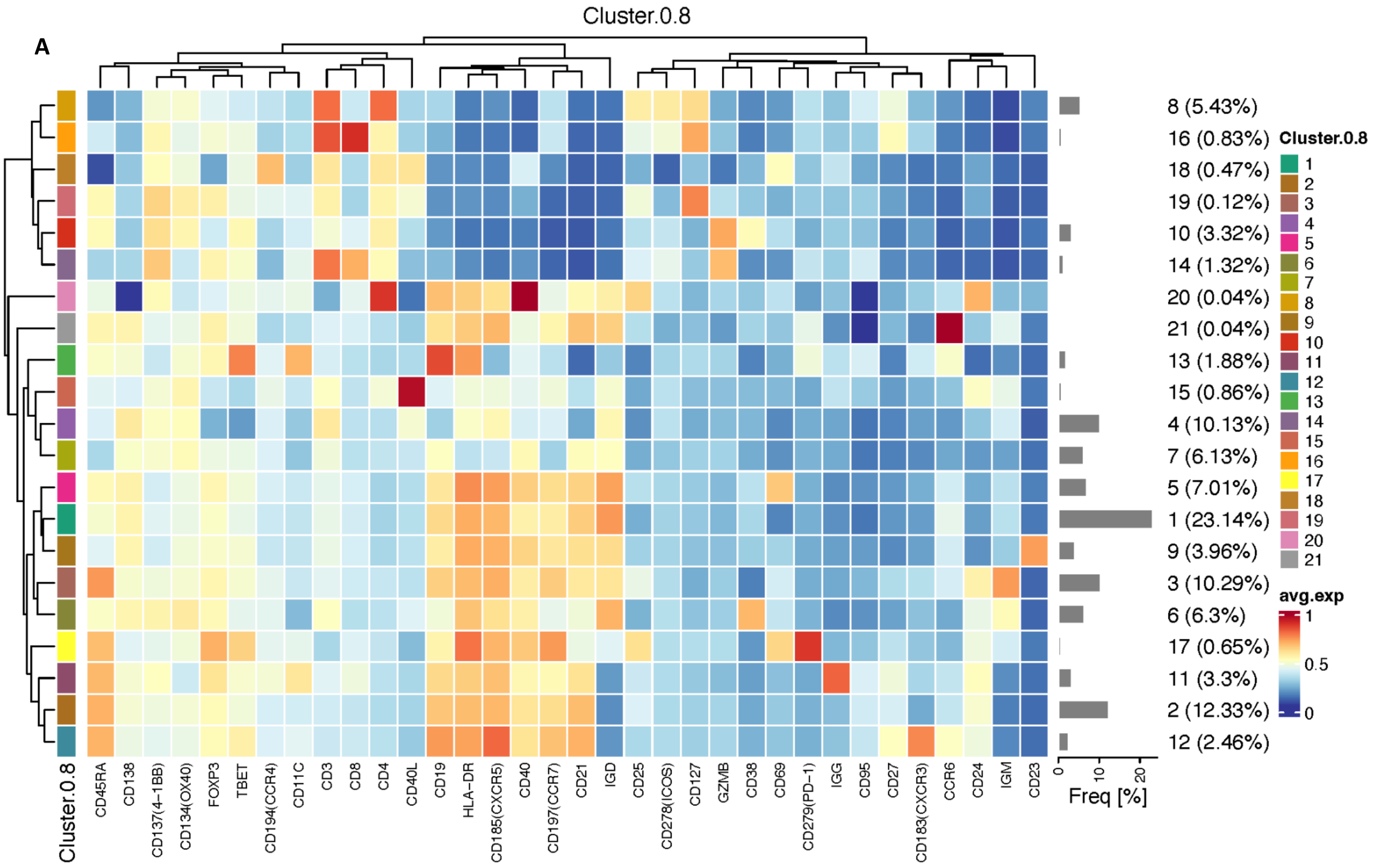

B

CD19+ phenotyping		
**Cluster ID**	**Phenotype**	**Antibody markers**
1	Naïve	CD19+ CD27- CD38 -/low CD24 low/mid IgD+ CCR7+
2	Switched memory (sM)	CD19+ CD27 mid CD38- IgM- IgD-
3	Naïve IgM+	CD19+ CD27- CD38 -/low CD24 mid/+ IgD+ IgM+ CCR7+
4	Naïve	CD19+ CD27- CD38 -/low CD24 low/mid IgD+
5	Activated Naïve	CD19+ CD27- CD38 -/low CD24 low/mid IgD+ CD69++ CCR7+ CXCR5+
6	Transitional	CD19+ CD27- CD24+ CD38+ IgD+
7	Naïve CXCR5- CCR7-	CD19+ CD27- CD38 -/low CD24 low/mid IgD+ CCR7- CXCR5-
8	Switched memory CD25+	CD19+ CD27+ CD38- IgM- IgD- CD25+ ICOS+
9	Naïve CD23+	CD19+ CD27- CD38 -/low CD24 low/mid IgD+ CCR7+ CD23+
10	CD19 low DN3	CD19 low CD27- CD38 -/low CD24 low/mid IgD- CD11c- CD21- GZMB+
11	IgG+ memory	CD19+ CD27+ CD38- CD24+ IgM- IgD- IgG+
12	Switched memory CXCR3+ CXCR5+	CD19+ CD27+ CD38- CD24+ IgM- IgD- IgG- CXCR3+ CXCR5+
13	DN2	CD19+ CD27- CD38 -/low CD24 low/mid IgD- CD11c+ CD21- Tbet+
14	DN3 GZMB+	CD19+ CD27- CD38 -/low CD24 low/mid IgD- CD11c- CD21- GZMB+
15	Naïve CD24+ CD40L+	CD19+ CD27- CD38 -/low CD24+ IgD+ IgM+ CCR7 mid CD40L+
16	EM1 CD8+ (from small number of CD3+ cells in CD19+ gate)	CD19- CD27+ CD3+ CD8+
17	DN1, activated-like phenotype	CD19+ CD27- IgD- CD24+ CD21+ PD-1++ CD69+ CXCR5+
18	Undefined cluster	CD19- CD3+ CD4+ CD69+ CCR4+
19	Not enough cells in cluster	16 total cells across all time points and treatments
20	Not enough cells in cluster	35 total cells across all time points and treatments
21	Not enough cells in cluster	34 total cells across all time points and treatments

Supplementary Figure 4

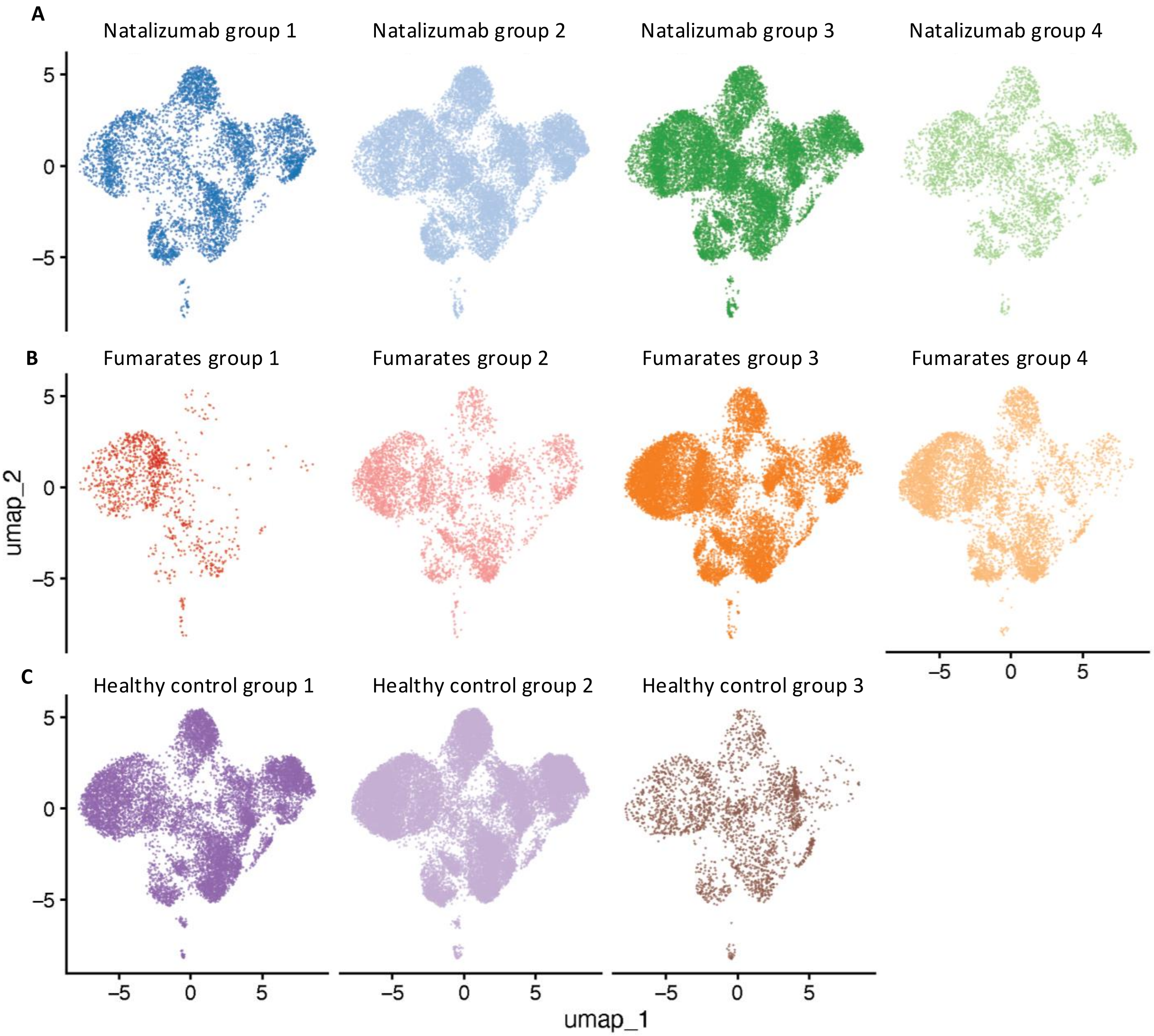

Supplementary Figure 5

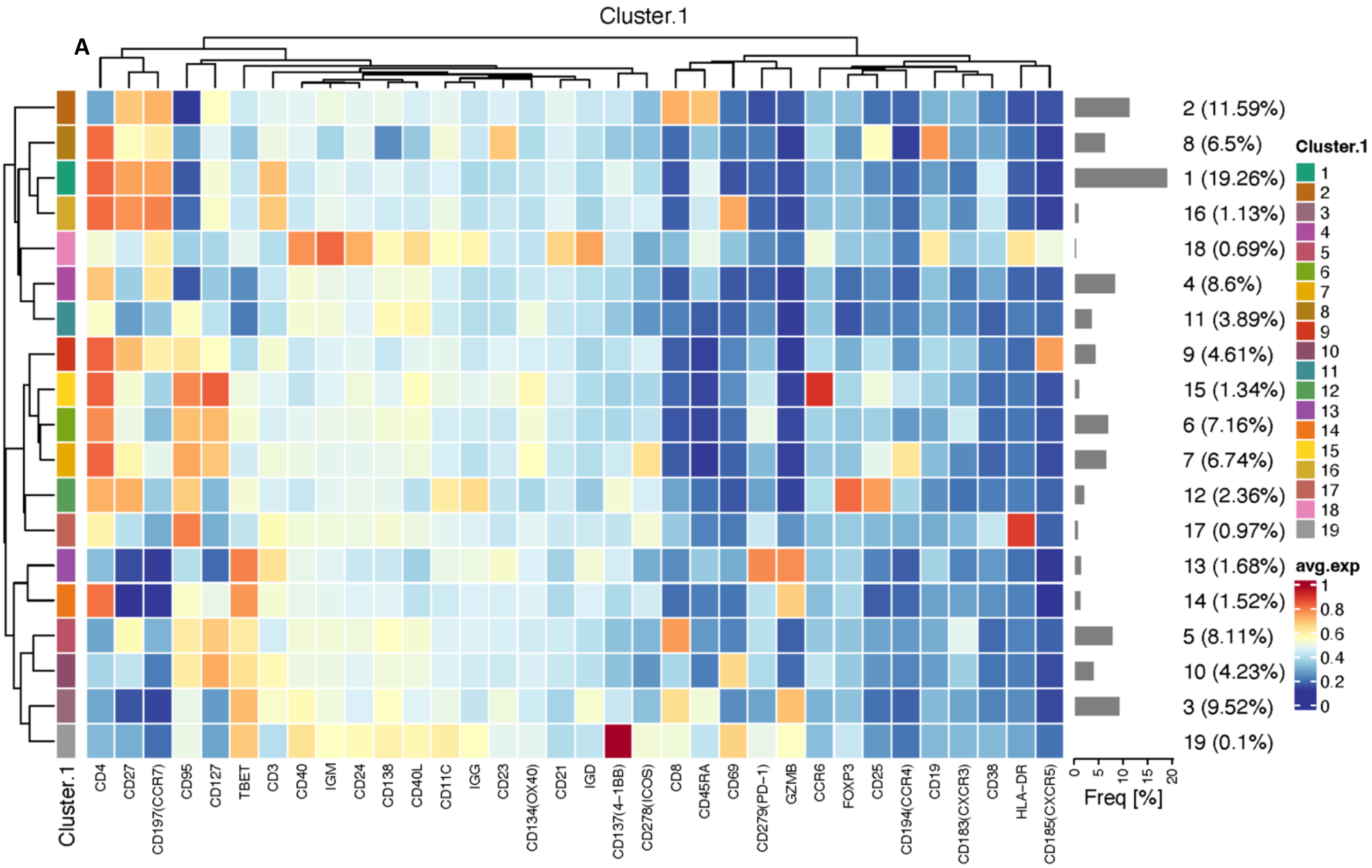

B

CD3+ phenotyping		
**Cluster ID**	**Phenotype**	**Antibody markers**
1	Naïve CD4+	CD4+ CCR7+ CD27+ CD45RA+
2	Naïve CD8+	CD8+ CCR7+ CD27+ CD45RA+
3	EMRA CD8+	CD8+ CCR7- CD27- CD45RA+ GZMB+ TBET+
4	EM2 CD4+	CD4+ CCR7+ CD27- CD45RA-
5	EM1 CD8+	CD8+ CCR7- CD27+ CD45RA- CD95+
6	EM1 Th1 like CD4+	CD4+ CCR7- CD27+ CD45RA- CD95+ CXCR3 mid
7	Th2	CD4+ CCR7 low CD27+ CD45RA- CCR4+ ICOS+
8	CM CD4+	CD4+ CCR7+ CD27+ CD45RA- CD25+
9	cTfh CD4+	CD4+ CCR7+ CD27+ CD45RA- CXCR5+ CD95+
10	EM1 CD8+	CD4 low CCR7- CD27- CD45RA- CD69+
11	EM3 CD4+ CD40L+	CD4+ CCR7- CD27- CD45RA- CD40L+ CD95 low
12	Treg CD4+	CD4+ CCR7- CD27+ CD45RA- FOXP3+ CD25+ CD127-
13	EMRA CD4 low PD-1+	CD4 low CCR7- CD27- CD45RA+ GZMB+ PD-1+
14	EM3 CD4+ GZMB+	CD4+ CCR7- CD27- CD45RA- GZMB+ TBET+ CD95+
15	Th17 CD27 mid CD95+	CD4+ CCR7- CD45RA- CD27 mid/low CCR6+ CD95+ CD127+
16	Activated Naïve CD4+ CD69++	CD4+ CCR7+ CD27+ CD45RA+ CD69++
17	EM3 CD4+ CD95++ HLA-DR++	CD4+ CCR7- CD27- CD45RA- CD95++ HLA-DR++
18	Not enough cells in cluster	CD19+ IgM+ IgD+ CD40+
19	Not enough cells in cluster	65 total cells across all time points and treatments

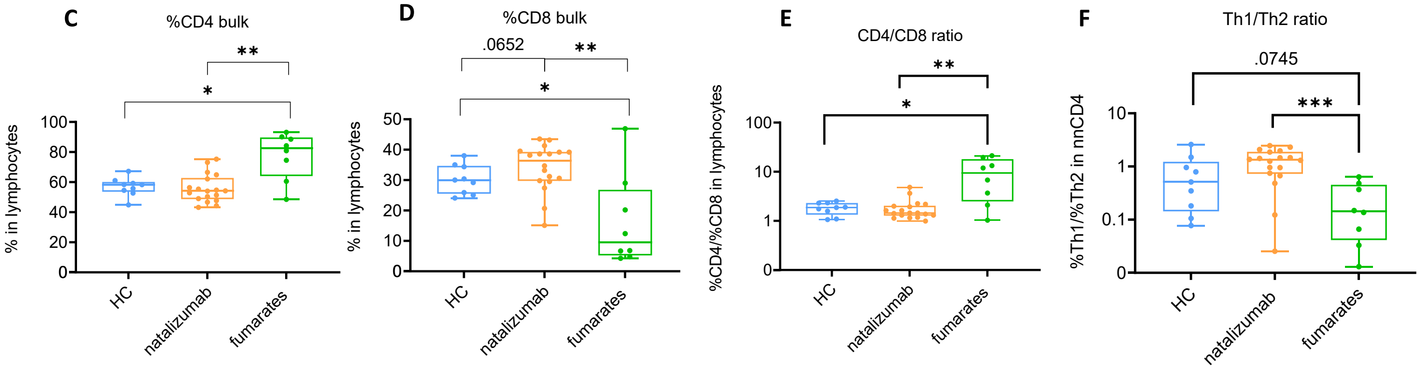

Supplementary Figure 6

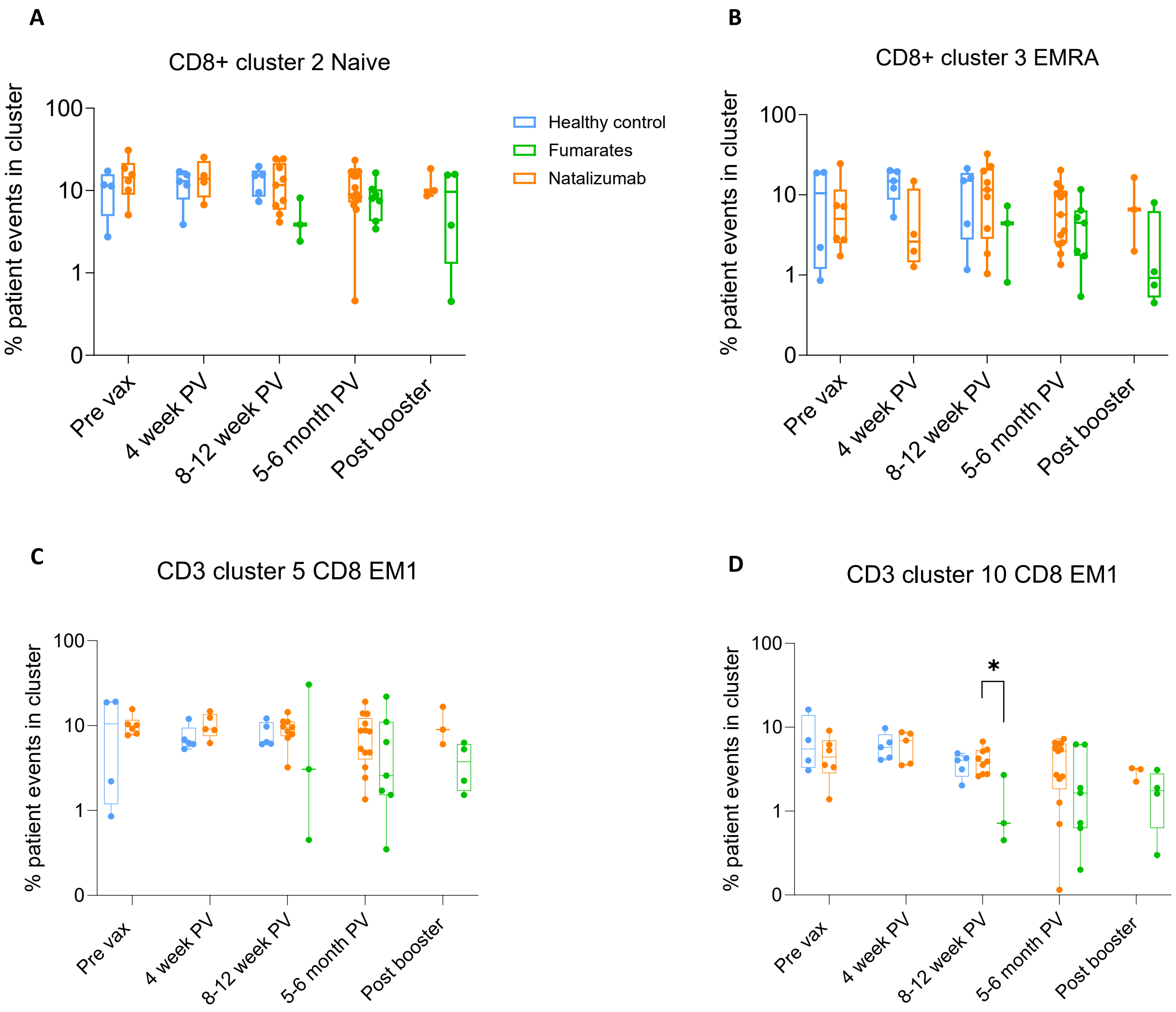

Supplementary Figure 7

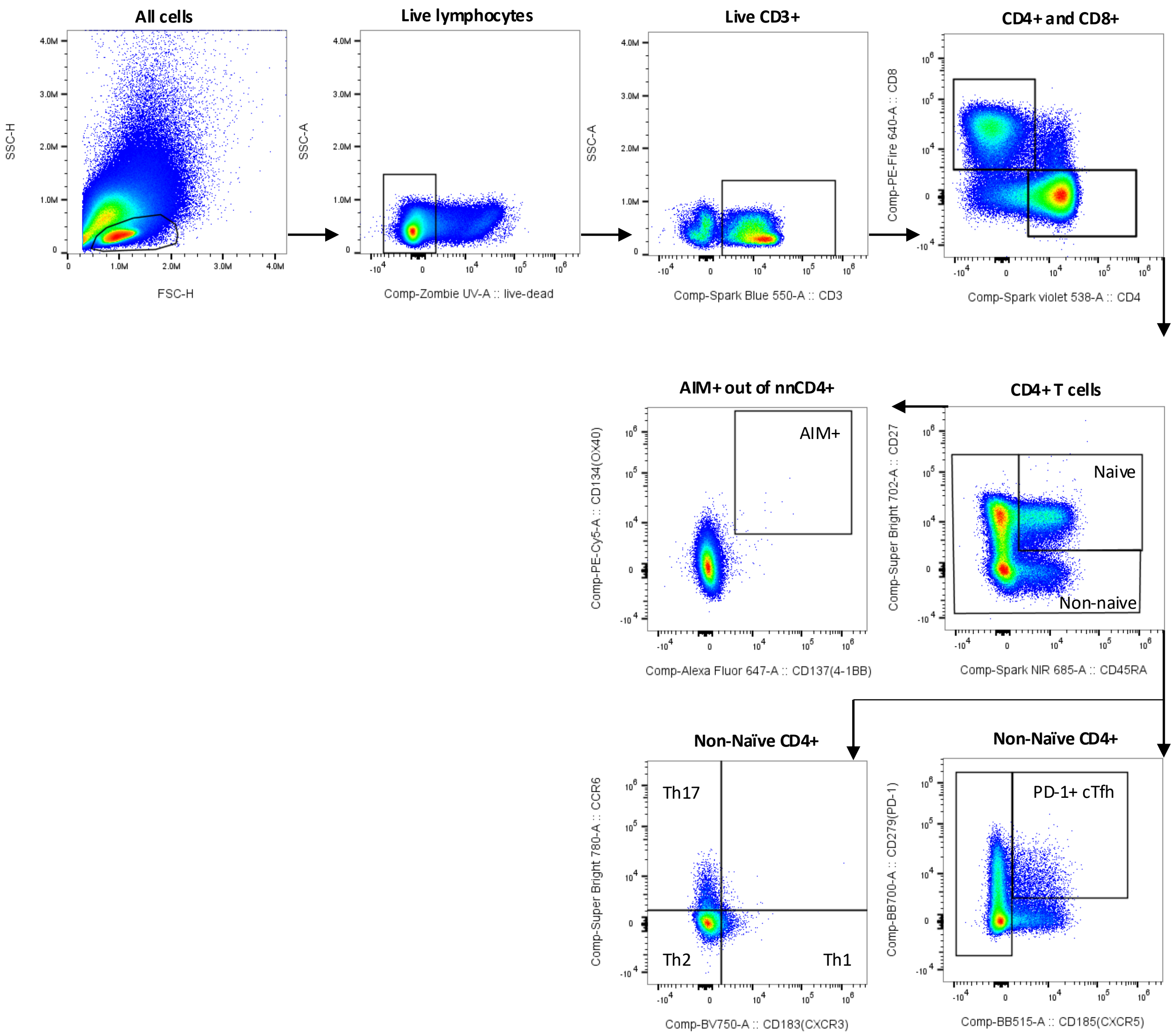

Supplementary Figure 8

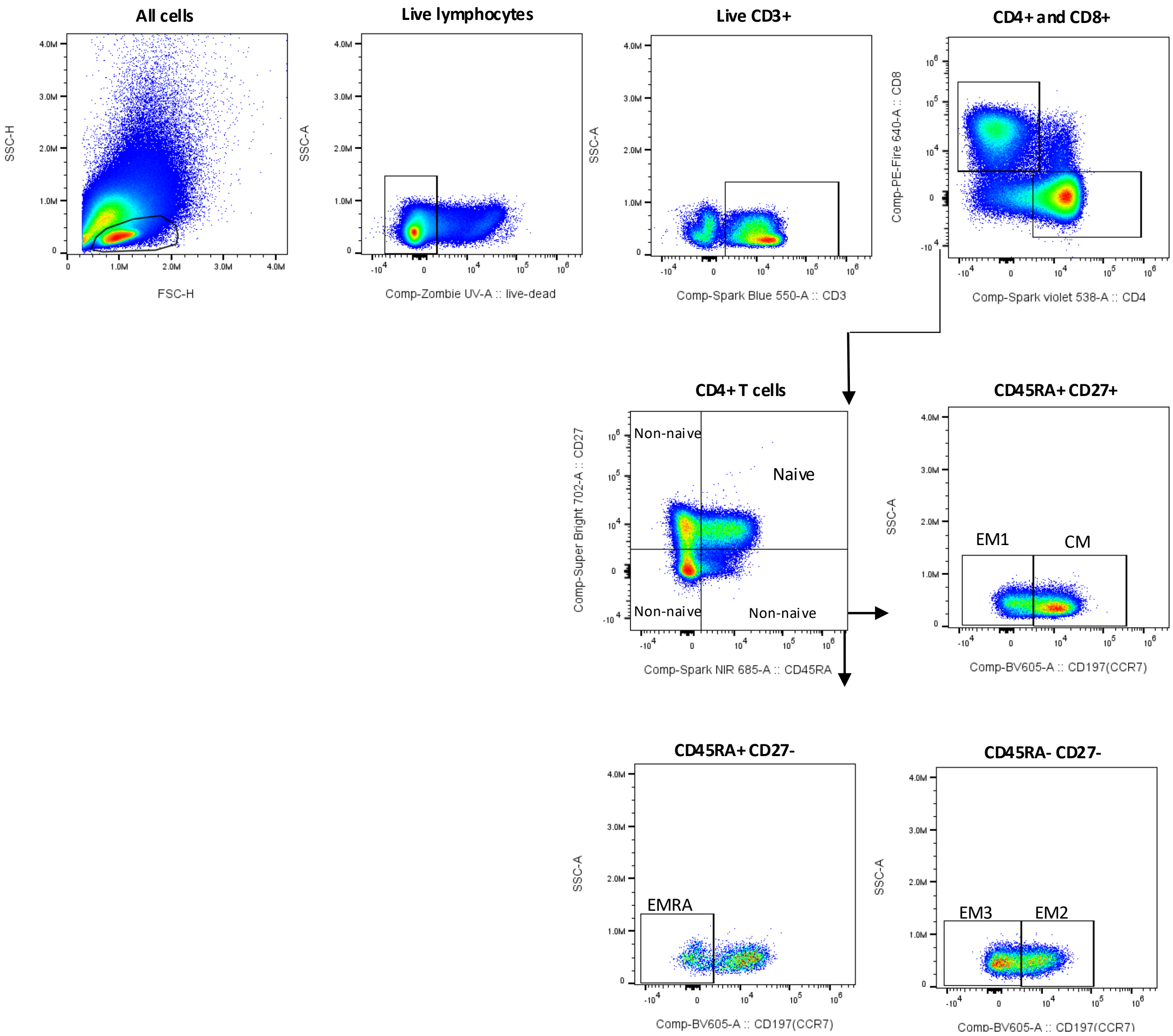

Supplementary Figure 9

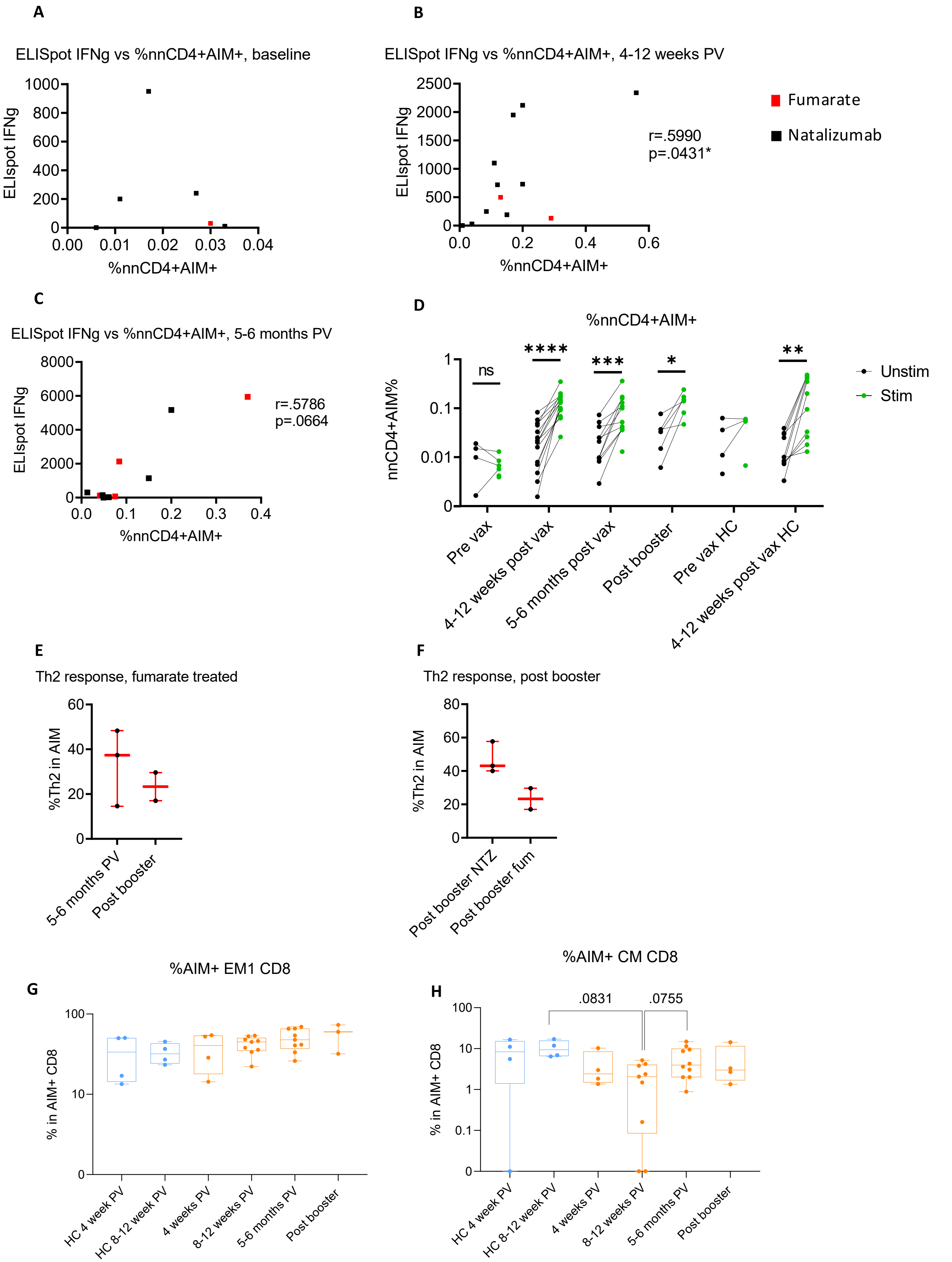

Supplementary Figure 10
